# Enhanced eMAGE applied to identify genetic factors of nuclear hormone receptor dysfunction via combinatorial gene editing

**DOI:** 10.1038/s41467-024-49365-z

**Published:** 2024-06-18

**Authors:** Peter N. Ciaccia, Zhuobin Liang, Anabel Y. Schweitzer, Eli Metzner, Farren J. Isaacs

**Affiliations:** 1 Department of Molecular, Cellular, and Developmental Biology, Yale University, New Haven, CT 06520 USA; 2 Systems Biology Institute, Yale University, West Haven, CT 06516 USA; 3 Physical and Engineering Biology, Yale University, New Haven, CT 06520 USA; 4 Department of Biomedical Engineering, Yale University, New Haven, CT 06520 USA; 5 Present Address: ZL: Institute of Molecular Physiology, Shenzhen Bay Laboratory, Shenzhen, 518132 China

**Keywords:** Synthetic biology, Synthetic biology, Genetic engineering

## Abstract

Technologies that generate precise combinatorial genome modifications are well suited to dissect the polygenic basis of complex phenotypes and engineer synthetic genomes. Genome modifications with engineered nucleases can lead to undesirable repair outcomes through imprecise homology-directed repair, requiring non-cleavable gene editing strategies. Eukaryotic multiplex genome engineering (eMAGE) generates precise combinatorial genome modifications in *Saccharomyces cerevisiae* without generating DNA breaks or using engineered nucleases. Here, we systematically optimize eMAGE to achieve 90% editing frequency, reduce workflow time, and extend editing distance to 20 kb. We further engineer an inducible dominant negative mismatch repair system, allowing for high-efficiency editing via eMAGE while suppressing the elevated background mutation rate 17-fold resulting from mismatch repair inactivation. We apply these advances to construct a library of cancer-associated mutations in the ligand-binding domains of human estrogen receptor alpha and progesterone receptor to understand their impact on ligand-independent autoactivation. We validate that this yeast model captures autoactivation mutations characterized in human breast cancer models and further leads to the discovery of several previously uncharacterized autoactivating mutations. This work demonstrates the development and optimization of a cleavage-free method of genome editing well suited for applications requiring efficient multiplex editing with minimal background mutations.

## Abstract (web summary)

Technologies that generate precise combinatorial genome modifications are well suited to dissect the polygenic basis of complex phenotypes and engineer synthetic genomes. Here the authors systematically optimize eMAGE for enhanced editing efficiency and editing distance and apply these advances to identify genetic factors of nuclear hormone dysfunction.

## Introduction

Genome editing has empowered researchers to elucidate causal links between genotype and phenotype, enact targeted genetic modifications to reprogram cellular behavior and design organisms with synthetic genomes^1–6^. Conventional genome editing technologies use programmable endonucleases, including zinc-finger nucleases (ZFNs), transcription activator-like effector nucleases (TALENs), and Clustered Regularly Interspaced Short Palindromic Repeats (CRISPR)-Cas nucleases, to generate site-specific DNA double-strand breaks (DSBs) in the genome. Subsequent DSB repair by endogenous machinery can introduce mutagenic gene disruptions via non-homologous end joining (NHEJ) or precise modifications via homology-directed repair (HDR)^4^. Recent advances in the expression of multiple guide RNAs and orthologous Cas nucleases have enabled multiplex CRISPR-Cas editing of 3–10 sites simultaneously^7^. In addition, several methods derived from CRISPR explore the fusion of nuclease-deficient Cas proteins to a variety of DNA effectors, such as deaminases^8,9^, error-prone polymerases^10^, transposases^11,12^ and reverse transcriptases^13^, to develop DSB-independent, alternative genome editing tools (e.g., Base editing^8,9^ and Prime editing^13^). However, for applications requiring the generation of multisite (>10) combinatorial genomic modifications with base-pair (bp)-level precision, conventional or modified CRISPR-based genome editing systems are inadequate due to inherent limitations^14^, such as off-target effects, cytotoxicity, editing accuracy, and multiplexing ceiling.

**Fig. 1:**
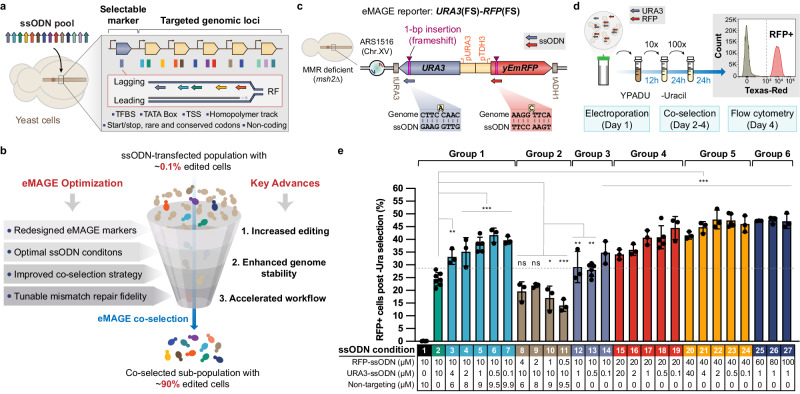
Increase eMAGE editing efficiency by redesigning co-selection markers and optimizing ssODN conditions. **a** eMAGE efficiently generates precise combinatorial genome editing in diverse genetic elements through directing the simultaneous annealing of multiple transformed ssODNs to the lagging strand of a replication fork. eMAGE co-selection via an ssODN-edited adjacent selectable marker enriches cells with combinatorial editing in the targeted genomic loci. RF: replication fork. **b** Systematic optimization of eMAGE at multiple levels by this study improves genome editing performance with a faster workflow and enhanced genome stability in eMAGE strains, resulting in ~90% edited cells in the co-selected population. **c** Fluorescent reporter design allows accurate and robust quantification of eMAGE editing efficiency. The yeast strain is deficient in MMR with deletion of the *MSH2* gene (*msh2*Δ). FS: frameshift. **d** Typical eMAGE workflow for co-selection and flow cytometry of yeast strains carrying the *URA3*(FS)-*RFP*(FS) reporter, taking 3.5 days from ssODN electroporation to RFP readout. **e** Comparison of eMAGE ARF across a series of conditions with different ssODN concentrations and ratios. All values represent mean ± SD for at least three independent biological replicates. p values of multiple-group comparisons from ordinary one-way ANOVA Dunnett’s test and p values of two-group comparisons from unpaired t-test. ns, not significant, *p < 0.05, **p < 0.01, ***p < 0.001. *p* = 1.4 × 10^−14^ for the optimized ssODN concentrations (condition 22).

Limitations in the scale and precision of current genome editing methods have motivated our recent work to develop a nuclease-independent eukaryotic multiplex genome engineering (eMAGE) platform^15^, which directs the annealing of multiple ~90-nt single-stranded oligodeoxynucleotides (ssODNs) at recombination intermediates to generate a set of precise combinatorial (10^5^−10^6^) genome modifications with up to 70% efficiency across many (10-100) loci in targeted genomic regions of *Saccharomyces cerevisiae* (Fig. 1a). Because of the relatively low recombineering efficiency (~10^−3^) of transformed ssODNs in the yeast genome, the eMAGE method uses a selectable marker (e.g., *URA3*) adjacent to the targeted genomic region to enrich ssODN-edited cells via a co-selection strategy^15,16^ (Fig. 1a and b). During the co-selection of eMAGE, yeast cells acquiring the defined mutation in the selectable marker can proliferate, resulting in a sub-population with high frequency of ssODN-mediated mutations in the adjacent genomic region. However, the original eMAGE co-selection design requires lengthy cell culturing with editing efficiency decreasing as the distance from the target mutation to the selectable marker increases. As with other ssODN-mediated genome editing methods^17–19^, eMAGE editing efficiency is greatly enhanced by inactivation of genes required for DNA mismatch repair (MMR)^15^. However, such constraints reduce genome stability, which could lead to the accumulation of unintended secondary mutations from unresolved DNA replication errors and spontaneous lesions, and requires prior modifications of the genome (e.g., deletion of MMR genes).

Polygenic mutations, involving multiple genetic loci, are implicated in a wide range of complex phenotypes and diseases such as cancer and neurodegenerative disorders. For example, mutations in the ligand-binding domains (LBDs) of nuclear receptors (NRs), such as estrogen receptor α (ERα) and progesterone receptor (PR), are frequently found in multiple types of cancers, including breast and prostate cancers^20–22^. A subset of such mutations confers constitutive receptor activation, which can promote uncontrolled cell growth and tumorigenesis. While a significant number of cancer-associated mutations have been identified in ERα and PR, most remain uncharacterized, and the potential for synergistic or synthetic interactions between mutations is largely unexplored. Conventional genome editing methods are limited in their ability to rapidly generate combinatorial mutations.

In this study, we describe several advances in eMAGE to address key limitations in the technology that constrain experimental time and editing efficiencies over large distances in more stable genomic strains (Fig. 1b) and apply these advances to study genetic factors implicated in nuclear hormone receptor dysfunction. First, we redesigned the eMAGE protocol and reporter editing assay while permitting the analysis of a larger number of edited cells. Second, we optimized ssODN concentrations, ratios, and modifications. Incorporating these modifications into a co-selection strategy led to a striking improvement in eMAGE editing efficiency and distance: we achieve up to ~90% edited cells (vs. 40%) in a population and ≥30% editing efficiencies across 20 kb (vs. 1%). Third, we developed a system for transient and controllable MMR suppression with dominant negative MMR mutants, achieving high-efficiency editing in DNA repair-proficient cells with enhanced genome stability and reduced frequency of unintended secondary mutations. Previously, mismatch repair knockout improved on-target editing efficiency at the expense of an elevated off-target mutational frequency. Finally, we applied the enhanced eMAGE editing method to generate precise individual and combinatorial genome modifications across the LBDs of ERα- and PR-derived synthetic transcription factors. We demonstrate that *S. cerevisiae* can be used as a model to study nuclear receptor dysfunction and identify previously uncharacterized single and combinatorial mutations that confer constitutive activity to either receptor-derived transcription factor. Advances of this study provide a genome editing method well suited to studying properties of biological systems that have complex genetic bases or that require high-efficiency multiplex editing, including the engineering of synthetic genomes.

## Results

### Optimization of co-selection workflow and ssODN conditions improves eMAGE editing efficiency

To enable rapid and combinatorial multiplex editing in a stable genetic background, we optimized the eMAGE methodology across four key areas: development of a dual reporter assay, ssODN optimization, dual marker co-selection, and conditional inactivation of DNA mismatch repair. We first set out to accelerate the eMAGE workflow by reducing co-selection time and increasing assay throughput. To measure eMAGE editing allelic replacement frequency (ARF), we previously used a *URA3*-*ADE2* reporter^15^ assay with a 5.5-day workflow to quantify *ADE2* ARF as the percentage of *ade2* edited cells (red colonies) on culture plates containing 5-fluoroorotic acid (5-FOA, a chemical compound that counterselects *ura3* cells). The redesigned eMAGE reporter harbors *URA3* and *yEmRFP* genes each carrying a predefined frameshift (FS) located in a previously characterized genomic locus^15^ of replication origin ARS1516 in chromosome XV (Fig. 1c). Yeast cells co-transformed with ssODNs restoring the correct reading frames of both *URA3* and *yEmRFP* were co-selected in liquid medium lacking uracil and analyzed via flow cytometry to determine the frequency of RFP positive cells, which represents eMAGE ARF (Fig. 1d). The *URA3*(FS)-*RFP*(FS) reporter provides three advantages over the previous *URA3*-*ADE2* reporter^15^. First, it reduces the experimental time needed for eMAGE co-selection from four to two days by making use of the more robust positive *URA3* selection and rapid RFP readout (Fig. 1d), avoiding laborious plating and colony screening procedures. Second, it eliminates interference from secondary mutations due to the extremely low frequency of spontaneous frameshift correction (which we did not observe), rendering co-selection more stringent by avoiding background 5-FOA resistant mutants (~10% of total *ura3* colonies^15^) that are unrelated to ssODN editing. Third, it facilitates rapid (~1 min. per sample) and more accurate quantification of eMAGE editing frequency via analyzing a larger population by flow cytometry (>10^5^ flowed cells vs. 10^2^−10^3^ colonies), resulting in a significantly improved assay throughput (>60 samples per hour). Together, this fluorescent reporter system permits convenient, fast, and accurate comparison of eMAGE ARF among diverse conditions, establishing a facile system for method development and optimization.

Using the fluorescent reporter system, we next attempted to optimize eMAGE ARF by investigating a wide range of concentrations between ssODNs targeting the edited locus (*RFP*) and the co-selection marker (*URA3*). Holding the concentration of the RFP-ssODN at 10 µM and total ssODN concentration at 20 µM by supplementing a non-targeting ssODN, we decreased the concentration of the URA3-ssODN and observed up to ~40% ARF in the *RFP* gene. This is a significant increase over the 25% baseline using the previous protocol^15^, in which ssODN concentration is 10 µM for both the co-selection marker (*URA3*) and editing targeted site (*RFP*) (Fig. 1e, group 1). Conversely, samples with a decreased RFP- to URA3-ssODN concentration ratio showed reduced eMAGE ARF in the range of ~13-22% (Fig. 1e, group 2). These results suggest that eMAGE ARF can be enhanced by limiting the cellular availability of ssODN targeting the selectable marker to increase the stringency of co-selection. With the same ratio of ssODN concentrations, the total concentration of ssODNs through 40 µM positively correlated with eMAGE ARF up to ~48% (Fig. 1e, compare groups 1, 3, 4, and 5). Further increase of RFP ssODN concentration to 60, 80, or 100 µM showed no further enhancement in eMAGE ARF (Fig. 1e, group 6), despite a slight increase in the RFP positive frequency in ssODN-transformed cells prior to *URA3* co-selection (Supplementary Fig. 1a), suggesting that eMAGE ARF reaches an efficiency ceiling with URA3-ssODN at ~40 µM. Given that concentration of URA3-ssODN also positively influences the initial number of URA3 cells (Supplementary Fig. 1b) and the total ssODN concentration negatively correlates with cell survival after electroporation (Supplementary Fig. 1c), we converged on optimal ssODN conditions: 20-60 µM total ssODN with a 20:1 ratio of ssODNs targeting edited site(s) to the co-selection marker (e.g., Fig. 1e, condition 22). We also attempted to increase the nuclease resistance of ssODNs by modifying their terminal nucleotides at both ends with phosphorothioate bonds and observed a minor, but statistically significant, ARF increase of ~5% over unmodified ssODNs at 40 and 80 µM (Supplementary Fig. 1d).

### Dual co-selection makers enhance multiplex editing and expand editing distance

**Fig. 2:**
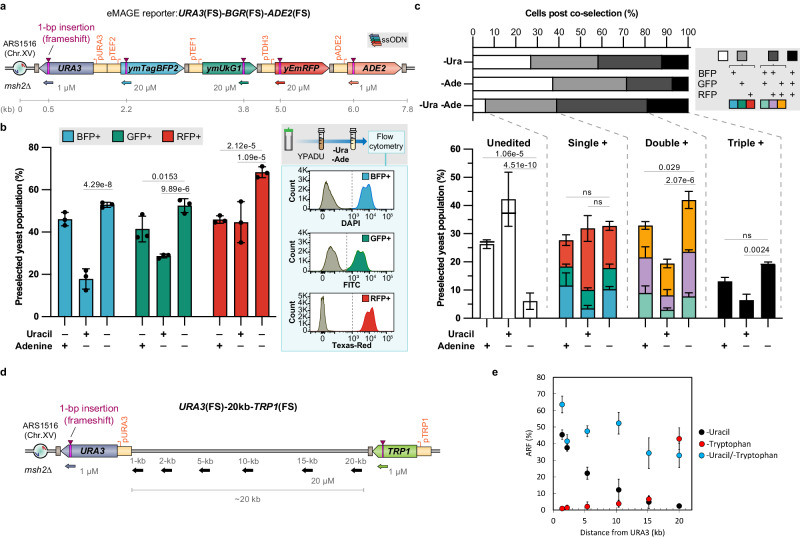
Improved eMAGE multiplex editing with dual selectable markers. **a** eMAGE reporter carrying three fluorescent and two selectable marker gene cassettes for rapid quantification of eMAGE multiplex ARF by flow cytometry. Sizes of the genetic elements are not shown in actual scale. The distance of frameshift mutations in each targeted gene to the replication origin is shown. **b** Comparison of eMAGE ARF of three fluorescent genes in cells co-selected using either single or dual selectable markers. Typical eMAGE workflow and representative flow cytometry plots of cells passed through -Ura -Ade co-selection are shown on the right. **c** Multiplex editing analysis of cells carrying the eMAGE reporter shown in (**a**). **d** 20 kb eMAGE reporter highlighting near-cognate EcoRI restriction sites targeted for editing (**e**). Effect of distance on ARF as a function of distance from selectable markers placed 20 kb apart. Co-selected cells have different combinations of fluorescent signals based on the frameshift correction status in three fluorescent reporter genes. The internal numbers of the lower stack bars represent mean percentages of the sub-populations with different fluorescent signal combinations. The upward-facing error bars on top of the stacked bars represent the SD of the mean percentages of the four editing categories (unedited, single-, double-, and triple-edited cells). The upward-facing error bars internal to the stacked bars represent the SD of the mean percentages of each sub-population with the specified multiplex editing status by the color codes. All values represent mean ± SD for at least three replicates. Statistical significance analysis of the means of each of the four editing categories was performed. p values from ordinary one-way ANOVA Dunnett’s test. ns, not significant, *p < 0.05, **p < 0.01, ***p < 0.001.

We previously observed that eMAGE ARF decreases as the distance between edited sites and the co-selection marker increases: ~40% at 1 kb, to ~15% at 5 kb, to ~ 5% at 20 kb^15^. We hypothesized that flanking the targeted genomic region with two co-selection markers would sustain high levels of multisite editing across a longer genomic locus. To test this hypothesis, we constructed a five-gene construct *URA3*(FS)-*BGR*(FS)-*ADE2*(FS), comprising three fluorescent reporters (*ymTagBFP2 – “B”*, *ymUkG1* – “G”, and *yEmRFP – “R”*) flanked by two selectable markers (*URA3* and *ADE2*), each of which bears a predefined frameshift mutation (Fig. 2a). Applying the ssODN concentration and ratio from the optimization experiments described above (1 µM for each co-selection marker and 20 µM for each fluorescent reporter), we observed that co-selection using both *URA3* and *ADE2* markers led to significantly higher eMAGE ARF (50-70%) in all three fluorescent reporters, as compared to co-selection using a single marker (Fig. 2b). Analysis of eMAGE multiplex editing further revealed that dual-marker co-selection enriched significantly more double- and triple-edited cells (~60% vs. ~30-40%) and dramatically reduced the unedited sub-population from ~30% to ~5% (Fig. 2c). ADE2 selection stringency was tested by plating liquid culture post-uracil/adenine dropout selection onto Complete Supplement Mixture (CSM) and scoring colonies for color. Red colonies were not observed, and white colonies were sequenced to confirm the ADE2 genotype. Furthermore, we explored the use of another auxotrophic marker *TRP1* as the second co-selection marker and observed similar editing efficiencies as *ADE2* (Supplementary Fig. 2). Using the three fluorescent genes in the eMAGE reporter, we further compared three genetic architectures for dual-marker co-selection-by-differentially gating the flow cytometry data to determine how the relative positions of the co-selection markers affect ARF (Supplementary Fig. 3a). We found that dual-marker co-selection in any placement configuration outperformed single-marker co-selection and that markers flanking the targeted site resulted in the highest ARF (Supplementary Fig. 3b).

To demonstrate that dual marker co-selection can expand the eMAGE editing distance, we constructed a strain in which TRP1(FS) was integrated 20 kb downstream of URA3(FS). The editing efficiencies of six previously tested sites were investigated, with distances 1.4, 2.2, 5.4, 10.4, 15.2, and 20 kb from ARS1516 in the original reporter strain (*URA3*−20kb). We observed that the dual marker selection can minimize the distance bias of using just one marker and maintain high levels of editing with a minimum of 32% editing and up to 63% (Fig. 2d).

### Tunable inactivation of DNA mismatch repair enables efficient eMAGE

**Fig. 3:**
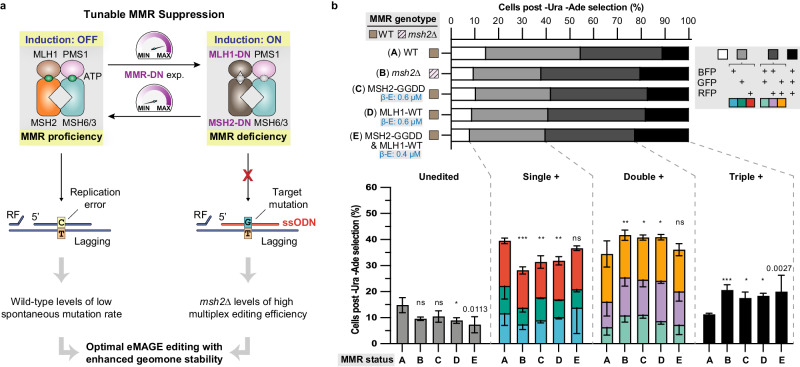
Transient inactivation of mismatch repair enables efficient eMAGE editing in DNA repair-proficient cells. **a** Rationally designed tunable overexpression of dominant negative subunits of the yeast DNA mismatch recognition and repair complex MutSα/β causes transient MMR deficiency in DNA repair-proficient cells, resulting in high on-target editing efficiency with low spontaneous mutation rate. MMR: DNA mismatch repair. DN: dominant negative. RF: replication fork. **b** Multiplex editing analysis of cells expressing selective MMR-DN mutants at three selective conditions. β-E: β-estradiol. The downward-facing error bars internal to the stacked bars represent the SD of the mean percentages of each sub-population with the specified multiplex editing status by the color codes. All values represent mean ± SD for at least three replicates. Statistical significance analysis between wild-type (WT) and each of the four MMR deficient strains, as well between *msh2*∆ and each of the three MMR-DN expressing strains was performed. p values from ordinary one-way ANOVA Dunnett’s test. ns, not significant, *p < 0.05, **p < 0.01, ***p < 0.001.

Consistent with prior observations^15,17^, we confirmed that deletion of *MSH2* (*msh2*∆) increased editing frequency three- to five-fold in all five markers of the multi-gene *URA3*(FS)-*BGR*(FS)- *ADE2*(FS) reporter after transformation of a pool of five ssODN at the optimal concentrations (i.e., 1 µM for *URA3*, *ADE2* and 20 µM for *B*-, *G*-, *RFP* genes) (Supplementary Fig. 4). This *msh2*∆ background also led to a 10-fold increase of double-edited URA3, ADE2 cells, bolstering the dual-marker co-selection strategy (Supplementary Fig. 4). To maintain high eMAGE ARF (~90% edited) while circumventing permanent MMR inactivation, we adopted a similar approach used successfully for MAGE in bacteria^23^: transient expression of dominant negative mutants of MMR proteins (MMR-DN) in DNA repair-proficient cells to temporarily suppress MMR prior to ssODN electroporation (Fig. 3a). To gain tighter control of the expression level of MMR-DN mutants and enhance the reproducibility of this method, we modified a previously reported β-estradiol inducible expression system^24^: we stably integrated the estradiol-responsive transcription activator (GEM) expression cassette in a defined genomic locus (YFL033C^25^) to constitutively express GEM at a moderate level in order to achieve tunable induction of MMR-DN upon β-estradiol titrations. We then characterized this regulator using RFP and observed β-estradiol-dependent high dynamic range inducible expression with near-linear dose response and no detectable uninduced expression. (Supplementary Fig. 5a). Addition of β-estradiol during growth and its removal during recovery results in transient MMR inactivation.

Next, we screened nine different MMR-DN mutants from three subunits (MSH2, MSH6 and MLH1) of the yeast mismatch repair MutS complexes, which have shown strong dominant negative effects in previous studies^26–28^, together with three wild-type subunit proteins (Supplementary Table 1), via episomal overexpression in a MMR-proficient strain (Supplementary Fig. 5b**)** harboring the *URA3*(FS)-*BGR*(FS)-*ADE2*(FS) reporter (Fig. 2a). Among the 12 tested MMR protein variants, moderate expression (β-estradiol: 0.4 µM) of MSH2-DN mutants^26^ led to the highest frameshift correction frequencies in three fluorescent genes, followed by MLH1-WT (for which overexpression was reported to have a DN effect^27^) and MLH1-DN mutants^27^, while expression of MSH6-DN mutants^28^ surprisingly showed no significant dominant negative effect in our assay (Supplementary Fig. 5c). Notably, we screened MMR-DN candidates using the frequency of fluorescent cells in ssODN-transformed populations prior to co-selection, which directly reflects the efficiency of ssODN incorporation and is thus more sensitive to MMR suppression than the post co-selection eMAGE ARF.

We then selected MSH2-GGDD and MLH1-WT for further optimization by fine-tuning the expression level of both mutants alone and in combination. Expression of MSH2-GGDD alone at relatively high levels (β-estradiol: 0.6–1 µM) or in combination with MLH1-WT at relatively low levels (β-estradiol: 0.2-0.4 µM) yielded similar frameshift correction frequencies as the *msh2*∆ cells with no statistically significant difference (Supplementary Fig. 5d). Importantly, analysis of eMAGE multiplex editing demonstrated that three selected MMR-DN mutant expression conditions each confer proportions of unedited, single-, double-, and triple-positive cells indistinguishable from *msh2*∆ background (Fig. 3b). It is important to note that optimization of ssODN conditions and the co-selection design alone improves upon our prior results^15^, albeit having fewer total edited cells than other strains with permanent or transient MMR inactivation.

Assessment of spontaneous mutation rate by Luria-Delbrück fluctuation analysis^29^ revealed that MMR-proficient cells carrying episomal MSH2-GGDD have a similar frequency of spontaneous mutations to wild-type cells, which can be transiently toggled to a level similar to *msh2*Δ cells upon MMR-DN expression (Supplementary Table 2, column A). Further analysis of data collected in this study demonstrates that tunable expression of MSH2-GGDD not only preserves wild-type levels of low spontaneous mutations (Supplementary Table 2, column B), but also conditionally enables *msh2*Δ levels of high-efficiency multiplex editing in a transiently relaxed genomic context with up to 10^4^ possible combinations of genomic diversity (Supplementary Table 2, column C-E). Notably, application of eMAGE often entails performing the workflow iteratively to drive multiple rounds of editing, which requires prolonged cultivation. By comparing the spontaneous mutation rate of CAN1 in strains that are *msh2∆* vs. MSH2 + p[MSH2-GGDD] ± estradiol, we demonstrate that inducible mismatch repair suppression leads to a mutation rate ~17x lower than mismatch repair knockout during 10-day cultivations (Supplementary Table 2, column B).

### Application of enhanced eMAGE to study cancer-associated mutations in the ligand binding domains of human estrogen and progesterone receptors

To leverage the strengths of genome editing via eMAGE, we sought to create a simplified model of the effects of mutations in the estrogen receptor alpha (ERα) and progesterone receptor (PR) ligand binding domains, which are frequently mutated in multiple types of cancers (e.g. breast, prostate cancer)^22,30–32^. ERα is a crucial transcription factor that regulates gene expression in response to estrogen. Most ERα mutations occur within the ligand binding domain (LBD), which is responsible for binding to estrogens and recruiting co-regulators. Similarly, PR is activated by progesterone, and frequent cancer-associated mutations occur in one of its two activating functions (i.e., AF2) located within its LBD.

We sought to develop a minimal model to study mutations in ERα and PR LBDs that confer constitutive nuclear import in the absence of ligands in a yeast system. In this model, estrogen- and progesterone-responsive synthetic transcription factors GEM and ZPM induce the expression of yEmRFP and ymUkG1 in response to the ligands β-estradiol and progesterone, respectively^24^. We chose this model for several reasons: first, synthetic transcription factors based on either of these NRs exhibit high dynamic range and no detectable basal activity. Second, although some cancer-associated alleles in either receptor LBD have been shown to confer constitutive activity in the absence of cognate hormone, most have not been characterized. Third, degenerate “NNK” (N = A, C, T, G; K = C, T) ssODNs enable full substitutional profiling and can target any sequence without constraints imposed by other genome editing methods like CRISPR HDR. Fourth, because eMAGE is well suited to multiplex editing, synergistic or synthetically interacting mutations can be introduced as easily as singleplex mutations.

**Fig. 4:**
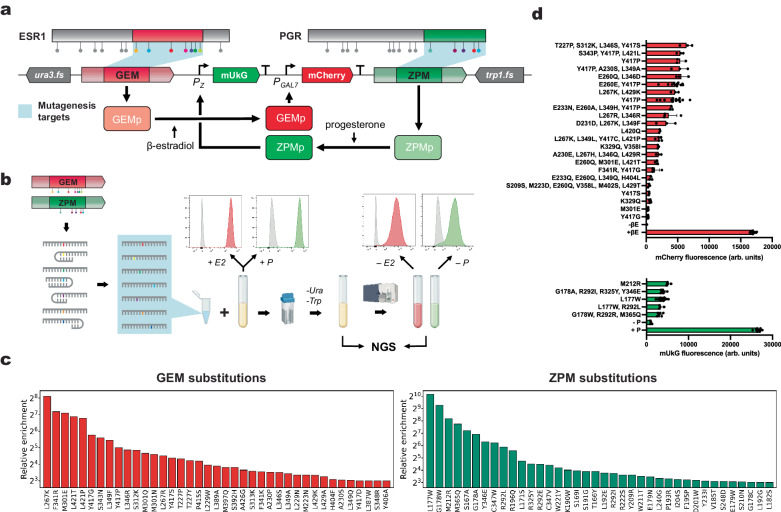
Characterization of GEM and ZPM transcriptional circuits at the eMAGE locus and identification of constitutively active variants via eMAGE. **a** Schematic representation of GEM and ZPM integrated at the eMAGE locus; GEM controls mCherry expression via Gal7 and ZPM controls mUkG expression via pZ. **b** workflow to diversify GEM, ZPM LBDs with degenerate ssODNs. **c** Read enrichment of GEM, ZPM variants between pre-sorted and post-sorted libraries, enriching for constitutively fluorescent variants. **d** Functional characterization of selected clones displaying constitutive transcriptional activity, compared to induced and uninduced wild-type controls. Data bars with identical amino acid substitution labels correspond to synonymous substitutions. Fluorescence values represent means ± SD of at least three independent biological replicates. β-E: β-estradiol; P: progesterone.

To develop this model, we integrated GEM and ZPM transcriptional circuits at the eMAGE locus (Fig. 4a). GEM controls the expression of Gal7-driven yEmRFP and ZPM controls the expression of pZ-driven ymUkG1. Gal7 was chosen over the Gal1/10 promoter to minimize homology within the eMAGE locus, as pZ is derived from Gal1/10. In addition, a terminator sequence of ADH1 was placed upstream of pZ to prevent its potential bidirectional transcription. We identified cancer-associated nonsynonymous mutations in the LBDs of either *ESR1* or *PGR* genes deposited in the cBioPortal for Cancer Genomics^33,34^. Informed by these data, we designed ssODNs encoding “NNK” mutations at positions corresponding to each mutation to minimize secondary structure (Supplementary Table 6), combined them into a pool with co-selection ssODNs, transformed the pool into the recipient, co-selected recombinants, and recovered constitutively active variants of either TF (Fig. 4b). Recovered populations were sequenced, revealing up to ~250-fold and ~1000-fold enrichment for GEM and ZPM variants, respectively (Fig. 4c). Enriched cells were plated, and the transcriptional activity of a subset of clones were characterized and compared to induced and uninduced WT controls (Fig. 4d). Enriched mutations were disproportionately represented among the characterized clones with constitutive transcriptional activity, confirming that NGS enrichment can read out on constitutive activity.

Among the sequenced variants, we observed both single and multiple mutations that conferred constitutive activity, which is generally lower than the ligand-induced controls. Intriguingly, several trends can be observed in these human ERα and PR mutations by using our yeast model with eMAGE diversification: first, substitutions of some amino acids are disproportionately amenable to driving dysregulation. GEM Y417 and E260 (i.e., ERα Y537 and E380), in particular, can be substituted to facilitate constitutive activation (Fig. 4d), consistent with several substitutions that have been previously reported to confer constitutive activity in cell culture, including ERα coordinates Y537S, Y537C, and E380Q^35,36^. These substitutions were identified in our library in clones isolated from RFP^+^ subpopulations, which either conferred constitutive activity alone or in combination with other substitutions. Among clones with multiple substitutions, cancer-associated alleles were disproportionately identified over other substitutions encoded through base degeneracy, suggesting that variants with multiple substitutions are still predictive of receptor dysfunction. Some variants with constitutive activity were identified in this screen that have not been observed in the clinic, e.g., Y417G, K329Q, and M301E in GEM (*ESR1* Y537G, K449Q, and M421E) and L177W and M212R in ZPM (*PGR* L721W and M756R). Y417G is notable, as the same residue when substituted with either serine or cysteine confers ligand-independent activation of ESR1 in mammalian cells^36^.

A majority of the clonally characterized variants in either receptor were multiply substituted. Some multiply substituted variants contained a substitution that alone was activating, like the presence of GEM Y417P in four multiply edited clones, whereas others contained unique combinations of substitutions. In some cases, multiply edited clones contain neutral and activating mutations: ZPM L177W R292L and L177W both confer the same level of constitutive activity. In another case, activating mutations appear additive: GEM K329Q was activating alone but resulted in further activation when combined with V358I. The presence of either of these types of variants demonstrates that this method can be used to dissect driver mutations from passengers, measure the phenotypic impact of each mutation, and identify synergistic activating substitutions.

## Discussion

In this study, we describe an enhanced and accelerated eMAGE genome editing method and applied these advances to study genetic factors implicated in nuclear hormone receptor dysfunction. From a technological perspective, this work improved on-target editing efficiency up to 70% for single edits and >60% for multiplex editing, resulting in ~90% of cells carrying intended genomic modifications after one round of eMAGE (Fig. 1b). A limitation in the original eMAGE editing method was the reduction in editing efficiency as a function of distance from the co-selectable marker. To address this limitation, we tested and validated a dual marker co-selection strategy, whereby the entire intervening sequence can be edited at efficiencies of ≥30% vs. ~1% with single marker co-selection (Fig. 2d). We speculate that this extended recombinogenic window is made accessible through a more stringent co-selection: by selecting for recombinants that have undergone recombination at both origin-proximal and origin-distal loci, recombinants in the intervening sequence are enriched through two additive polar effects.

We sought to reduce the background mutation rate in strains adapted for eMAGE editing using an analogous approach employed in MAGE: transient MMR suppression. When an ssODN with an encoded mutation is incorporated into the genome, a mismatch is created that is either corrected through MMR or that otherwise clonally segregates in a subsequent round of replication. To enable eMAGE editing with MMR- efficiencies in MMR^+^ strains, we overexpressed WT or mutant alleles of three MMR proteins in *S. cerevisiae*: MSH2, MSH6, and MLH1. WT MLH1 overexpression and mutant MSH2 and MSH6 overexpression were previously reported to suppress MMR. Surprisingly, we found that overexpression of dominant negative MSH6 alleles did not affect genome editing efficiency in an experiment in which incorporated ssODNs created 1 bp frameshifts, a substrate of Msh2-Msh6 heterodimers. This result could be explained by an additional role that MSH2 and MSH6 play in genome maintenance: heteroduplex rejection during homologous recombination^37,38^. eMAGE depends on the annealing activity of Rad52, which may be affected by ssODN heterology in a Msh2/Msh6-dependent manner in Rad52-dependent recombination pathways^15^. This explanation is supported by the finding that, unlike heteroduplex rejection, MMR is mostly inactive during G2/M in *S. cerevisiae*^39^. Likewise, the annealing activity of Rad52 is constrained to G2/M^40^. These observations suggest that ssODNs anneal post-replicatively via Rad52, and that rejection of their encoded heterology can be suppressed through a subset of dominant negative MMR alleles that also have dominant negative heteroduplex rejection activity. These observations will guide future work in identifying the precise mechanisms underlying the improvements in editing efficiency attributable to MMR proteins.

Leveraging these advances, we were interested in determining whether genome editing via eMAGE would enable high throughput screening for activating mutations in human nuclear receptor-derived transcription factors. We mutated the LBDs of ERα - and PR-derived transcription factors in a pool to simultaneously study uncharacterized cancer-associated mutations as well as to identify activating mutations yet to be identified in patient samples. We observed the enrichment of a subset of mutations in either receptor LBD, which we followed up with clonal characterization. Clonal characterization revealed three classes of substitutions that conferred constitutive activity: single substitutions described in the literature (ESR1 Y537S, Y537C, and E380Q), and previously unreported single and multiplex substitutions. That single substitutions described in the literature were enriched in NGS and were constitutively active in yeast demonstrated that this approach could be predictive of uncharacterized substitutions, such as those identified in this screen. Multiply substituted isolates sometimes encode mutations identified in singly substituted isolates, suggesting that bystander mutations only partially explain constitutive activity. Moreover, multiply substituted isolates reveal synergistic activating mutations. Neither of these combinations of substitutions nor a subset of individual substitutions have previously been identified in the clinic. This could be due to the limited availability of genetic information on patient samples and to the types of mutations required to confer some of the substitutions identified here: many ssODNs in this library introduce two or three mutations in order to access all amino acids for a given target, which are less likely to occur through natural mutation.

Utilizing enhanced eMAGE, we demonstrate a model to study nuclear receptor dysfunction in *S. cerevisiae*. We anticipate this model to be used not only to study ESR1 and PGR dysfunction but also to interrogate recently described NR-derived synthetic transcription factors: TFs derived from the human mineralocorticoid and androgen receptor LBDs could be targeted for combinatorial mutagenesis via eMAGE to study dysfunction in the receptors they derive from, such as early onset hypertension^41^ and prostate cancer^42^. Furthermore, we anticipate that the previously unknown mutations described here could be the focus of future experiments in cell culture to further interrogate their dysfunction.

We expect that these advances will empower eMAGE for tasks that demand precise and combinatorial diversification of other targeted genetic elements with prolonged cultivation^43^, such as protein engineering, molecular evolution, heterologous pathway optimization, and synthetic genome construction^1^. Each of these applications will also benefit from facilitating multiplex editing at distal loci or on separate chromosomes, which is currently limited by this method and a key goal of future work. Given the highly conserved nature of eukaryotic DNA replication and repair, we envision that the reported advances of eMAGE can guide the subsequent development of analogous nuclease-independent genome editing technologies in other yeast and multi-cellular eukaryotes.

## Methods

### Strain construction

Yeast strains used in this study can be found in Supplementary Table 1 and sequences of important genomic loci can be found in Supplementary File 1. To construct the yeast strain harboring the *URA3*(FS)-*RFP*(FS) eMAGE reporter (Fig. 1c), the *URA3* cassette of EMB101^15^ was first replaced by a PCR product of *ymUkG1*^44^(FS)-*yEmRFP* ^45^(FS) cassettes via standard homologous recombination (HR) and 5-FOA counterselection, resulting in yeast strain SZL149. Next, a *URA3* cassette was reintroduced to replace the *ymUKG1*(FS) cassette via HR and uracil drop-out selection, resulting in strain SZL238 with *URA3*-*yEmRFP*(FS). Next, a frameshift mutation was introduced in the *URA3* coding sequence by eMAGE with an ssODN, followed by 5-FOA counterselection, resulting in strain SZL247. Finally, the *MSH2* gene was deleted by a *KanMX4* cassette conferring G418 (geneticin) resistance, resulting in strain SZL335 that was used in Fig. 1e. To construct strains capable of β-estradiol inducible expression, GEM effector expression cassette together with the *LEU2* marker was PCR amplified from pHES839^24^ (Addgene # 87941) and integrated downstream of the *YFL033C* locus of SZL149 and SZL281 (SZL149+*msh2*Δ), resulting in strain SZL281 and SZL308, respectively. To construct the yeast strains harboring the *URA3*(FS)-*BGR*(FS)-*ADE2*(FS) eMAGE reporter (Fig. 2a), *URA3*-*ymTagBFP2*(FS) cassettes (*ymTagBFP2*: codon-optimized *mTagBFP2* ^46^ for *S*. *cerevisiae* from this study, coding sequence can be found in Supplementary File 1) was integrated between ARS1516 and the *ymUkG1*(FS) cassette of SZL281 and SZL308, resulting in strain SZL345 and SZL347, respectively. Next a frameshift mutation was introduced in the *URA3* and *ADE2* coding sequences by a round of eMAGE with ssODN OZL327 and OZL428, resulting in strain SZL376 that was used in Fig. 3b and Supplementary Fig. 3c, d (MMR-WT), and strain SZL348 that was used in Figs. 2b, c, 3b and Supplementary Fig. 3c, d (*msh2*Δ). Yeast transformations were performed via the optimized PEG/lithium acetate protocol of the Wilson lab^47^. Briefly, 10 ml of YPAD or YPADU were inoculated with 200 µl of overnight culture of the strain to be transformed and were grown 3–5 hours until OD_600_ = 0.5 − 0.8. Cells were washed once in 10 ml of 100 mM LiAc and resuspended in 200 µl 100 mM LiAc for 10 minutes. During this time, 10 µl/transformation of freshly boiled single-stranded salmon sperm DNA (ThermoFisher catalog 15632011) was mixed with 1–20 µl DNA to be transformed. 100 µl cells were added to and vortexed with this mixture. Separately, 560 µl 50% w/v PEG 3350 (pH 7.5) (Spectrum Chemical catalog PO125500GM), 70 µl 1 M LiAc, and 70 µl 10x TE were mixed per transformation. The PEG mix was added to each cell suspension, was vortexed, and was incubated at 30 °C for 30 min. 85 µl dimethyl sulfoxide was added to each transformation and mixed well. Suspensions were heat shocked at 42 °C for 7 min. and then allowed to rest at room temperature for 2-3 min. Cells were washed 3 times in 1 ml sterile water and were plated on appropriate media. All eMAGE reporter loci were sequence verified in the final strains.

### Media and culturing conditions

Unless otherwise stated, yeast strains were grown at 30 °C in a roller drum (Fisher Scientific, catalog 14251- 228Q/ 232Q) incubator. For yeast cultivation, cells were grown in a nonselective YPADU liquid medium, which consists of YPD (10 g/l Yeast Extract, 20 g/l Peptone, 20 g/l Dextrose), supplemented with 40 mg/l adenine hemisulfate, and 20 mg/l uracil. For auxotrophic selection, synthetic defined media with 2% glucose and drop-out of corresponding nutrients were used [Sunrise Science, MP Biomedical]. For eMAGE with frameshifted markers, yeast was cultured in YPADU and recovered immediately after ssODN electroporation in YPADU with 0.5 M sorbitol. For culturing yeast strains carrying episomal expression vectors of MMR genes, YPADU with 200 µg/ml zeocin was used.

### Plasmid cloning

Plasmids used in this study can be found in Supplementary Table 2. For inducible expression of MMR subunits, the *LEU2* cassette of pRSII425^48^ (Addgene #35468) was replaced by a Sh *ble* cassette conferring zeocin resistance, resulting in a shuttle vector pRSII42B (this study) harbored in strain SZL134. Wild-type MMR genes (*MSH2*, *MSH6*, *MLH1*) were PCR amplified from genomic DNA of yeast strain BY4741^49^ and cloned together with the bidirectional inducible *Gal1-10* promoter into the PCR amplified pRSII42B backbone via Gibson assembly^50^ using NEBuilder Kit (NEB, catalog E2621). MMR-DN mutant genes were subsequently generated via site-directed mutagenesis using either Q5 Site-Directed Mutagenesis Kit (NEB, catalog E0554S) or QuikChange Lightning Multi Site-Directed Mutagenesis Kit (Agilent, catalog 210515) according to the manufacturer protocols.

### ssODN electroporation

ssODN electroporation was carried out using the previously reported protocol^15^. Briefly, a single colony was inoculated in a 2 ml starter culture of appropriate media and grown overnight to saturation. The next day, the culture was diluted 1:100 into 10 ml fresh media and grown until the OD600 is between 0.6 and 1.0 in 4-6 hours. Cells were then pelleted and incubated in DTT-LiTE buffer (100 mM lithium acetate, 25 mM DTT, 500 mM hydroxyurea, 1x TE at pH 8.0) for 30 min at 30 °C in a roller drum. Cells were then washed with ice-cold water and 1 M sorbitol. The specified combinations and concentrations of ssODNs were prepared in 200 ul 1 M sorbitol, and used to resuspend the washed cells. ssODN electroporation was carried out in a 2-mm cuvette (Biorad, catalog 1652086) at 1500 V, 25 µF, 200 Ω. Immediately after electroporation, 1 ml eMAGE recovery medium (YPADU with 0.5 M sorbitol) was added to the cuvette, and the resuspended cells were added to a 15 ml culture tube containing 4 ml recovery media (final volume: 5 ml). Cells were then incubated at 30 °C in a roller drum for 12 hours prior to co-selection. Information of ssODNs used in this study can be found in Supplementary Table 3.

### eMAGE co-selection

After overnight recovery after ssODN electroporation, 500 µl of saturated culture was spun down, washed once with 1 ml sterile water, and transferred to 5 ml synthetic defined media with drop-out of auxotrophic nutrients based on the applicable selectable markers. This 10-fold diluted culture was first grown for 24 hours (single-marker co-selection) or 48 hours (dual-marker co-selection), then diluted 100-fold in 3 ml of the same synthetic defined media and incubated for another 24 hours to saturation. Sequential dilutions of 10- and 100-fold in two steps ensure that unedited cells represent less than 0.1% in co-selected population and therefore exert negligible influence on eMAGE ARF quantification, while making sure sampling errors in each dilution are within acceptable range (95% confidence level and 2% margin of error).

### Flow cytometry analysis

Flow cytometry was carried out on BD FACSAria II. ymTagBFP2 was excited by the violet laser (405 nm) with the DAPI emission filter (450/40); ymUkG1 was excited by the blue laser (488-nm) with the FITC emission filter (530/30); yEmRFP was excited by the green laser (561 nm) with the Texas-Red emission filter (610/20). Yeast cells were washed and resuspended in 1x PBS prior to flow cytometry. Flow cytometry data were analyzed in FlowJo V10.

### eMAGE editing with markers placed 20 kb apart

Ura3(fs) and trp1(fs) markers were placed 20 kb apart from one another on Chr XV. ssODNs were designed to target the intervening sequence at loci that had near-cognate EcoRI recognition sequences. In this scheme, incorporation of an ssODN introduces a 1-nucleotide mutation that creates an EcoRI restriction site. eMAGE co-selection was performed as described above using these ssODNs, and populations were selected in either -Ura, -Trp, or -Ura/-Trp conditions. Post-selection, each ssODN target site was amplified for at least 48 clones. The amplicons were digested with EcoRI and run on agarose gels. If a single amplicon of the correct length was observed, the site was scored as having not been edited. If two bands were observed of expected lengths, the site was scored as having been edited. Means and standard deviations were calculated from three replicates.

### Cell survival assay

To measure the effect of ssODN concentration on cell survival, strain SZL335 was prepared the same way as in eMAGE and electroporated with representative concentrations of RFP- and URA3-ssODN. Immediately after ssODN electroporation, cells were plated onto YPADU plate with 0.5 M sorbitol and incubated at 30 °C for 2 days. The number of colonies on each plate was counted and normalized with the plating volumes and dilution factors to calculate the percentage of viable cells in each condition. Cell viability data were normalized to untreated cells (100 % viable).

### β-estradiol inducible expression

The β-estradiol inducible system was constructed as above (Strain construction and Plasmid cloning). Constitutively expressed GEM translocates from cytosol to nucleus upon β-estradiol binding and activates transcription of the *Gal1-10* and *Gal7* promoters. β-estradiol (Sigma, catalog E1024) was prepared as a 10 mM stock solution in 100% ethanol, then diluted in media to working concentrations between 0 and 1 µM. Titration of inducible expression levels was empirically determined using RFP expression. Plasmids were maintained via zeocin selection. RFP fluorescence and cell density (OD600) were measured in each β-estradiol concentration along a 24-hour time course using a BioTek plate reader (Synergy H1), and selective time points were plotted to visualize the dose-response curve of gene expression as shown in Supplementary Fig. 4a.

### Fluctuation analysis

To quantify the spontaneous mutation rate of selective eMAGE strains in Fig. 3d, Luria-Delbrück fluctuation analysis was performed using a protocol modified from Lang^29^. In brief, a 2 ml starter culture was inoculated from a single colony in synthetic defined medium with drop-out of uracil and grown overnight to saturation. The next day different cultures were adjusted to the same OD of 1.0 and then diluted 5,000-fold in nonselective YPADU medium without or with β-estradiol in the specified concentration. 100 µl of diluted yeast culture was added to each well of a 96-well plate at a seeding density of 200–600 cells and incubated at 30 °C without shaking for 2 days until saturation. For each 96-well plate, cultures of 12 wells were pooled and used to determine the average number of cells per culture (**N**) by limited-dilution plating. The entire cultures (100 µl) of the remaining 84 wells were spot-plated on 5-FOA media and incubated at 30 °C for 4 days. Mutation rate (**µ**): mutation events per cell per division or generation, was calculated using the *p*0 method with formula: µ = −ln(*p*0)/N, where ***p*0** is the fraction of spot cultures with no colony.

### Calculations of spontaneous mutation and eMAGE on-target editing

In Supplementary Table 2, spontaneous mutation rates of the *URA3* gene shown in column A were determined by the fluctuation analysis described above. Estimated numbers of spontaneous mutations shown in column B were calculated by normalizing the size of the *URA3* gene (804 bp) to S288c genome (12 mb) and multiplying the numbers of cell division in a typical round of eMAGE (~66 cell divisions) or after accumulative cultivation of 10 days (~120 cell divisions). The cell division numbers were estimated based on the total time of yeast cultivation (i.e., ~132 hours during eMAGE comprising 48-hour: isolated colony forming from frozen glycerol stock, 24-hour: eMAGE starter culture incubation, 12-hour: recovery post ssODN electroporation, and 48-hour: eMAGE co-selection) with an average 2-hour doubling time. The Average numbers of eMAGE on-target edits shown in column C were derived from data of the eMAGE multiplex editing experiments shown in Fig. 3b using formula: Avg. edits= Σ[(% of sub-population)×(number of edits)], where ‘% of sub-population’ is the percentage of cells with either 0, 1, 2 or 3 edits. The number of double-edited URA3, ADE2 cells after ssODN electroporation and recovery shown in column D were calculated with cell viability data (~10% at 60 µM ssODN) shown in Supplementary Fig. 1c and the *URA3*, *ADE2* double frameshift correction frequency (~0.0015% for WT, ~0.015% for *msh2*Δ and MSH2-GGDD (β-E: 0.6 µM)) shown in Supplementary Fig. 3. Total numbers of eMAGE edited sites shown in column F were calculated by multiplying the values in column C and D.

### Dual hormone biosensor

The dual hormone biosensor was constructed by assembling eight PCR products and a CEN4/HIS3 yeast artificial chromosome (YAC) backbone using transformation-assisted recombination (TAR)^51^. Cells were plated on CSM ‑histidine. After three days, colonies were screened for correct assembly and inoculated into CSM -histidine. YACs were purified from overnight cultures using Zymoprep Yeast Plasmid Miniprep II [Zymo] and were transformed into TransforMax EPI300 competent *E. coli* [VWR]. Clones were picked and sequence-verified. The resulting plasmid, pPCY359, was linearized with BstEII and SrfI, transformed into sZL247, and plated on CSM -uracil. URA3 and ADE2 homology encoded in the linearized YAC provided the homology to direct the cargo to the eMAGE locus. Clones were screened for correct integration using colony PCR spanning either terminus of the eMAGE locus. To prepare the strain for dual marker co-selection, the URA3 marker used to select for and direct the linearized plasmid to the eMAGE locus was frameshifted again with eMAGE, in which clones were selected as described above using 5-FOA. The resulting had a *ura3*/*trp1* genotype, ready for diversification with eMAGE.

Genome editing via eMAGE was conducted as described above, with the following modifications: ssODNs targeting URA3 and TRP1 for frameshift correction were supplied at 10 µM, and the hormone biosensor-targeting ssODN pool was supplied at 40 µM. The ssODN pool was prepared by mixing equimolar amounts of each degenerate ssODN listed in Supplementary Table 6 (ssODN design described below). In this application, since both GEM and MMR-DN are induced by β-estradiol, transient MMR inactivation was not emploed. After recovery and co-selection, RFP+ and GFP+ cells were separately recovered with a BD FACSAria II. The sorted populations were expanded overnight in CSM complete. gDNA was purified using Qiagen DNeasy Blood & Tissue kit following the yeast supplementary protocol. PCR products spanning either LBD were amplified from gDNA using NEBNext® Ultra™ II PCR mix and were sequenced by collecting 150 bp paired end reads on an Illumina MiSeq instrument [Massachusetts General Hospital].

### Computational design of ssODNs

ssODNs were designed using a script to minimize oligo secondary structure. Briefly, each ssODN was designed to replace a selected codon with a degenerate “NNK” sequence, flanked by homology on either end. To minimize secondary structure, a sliding window was applied to each ssODN, where the length of the upstream and downstream homology arms were incrementally adjusted while maintaining the total ssODN length at 90 nucleotides. At each increment, the minimum free energy was calculated using ViennaRNA^52,53^. The window with the highest folding energy was used for each ssODN. ssODNs used to diversify the dual hormone biosensor are listed in Supplementary Table 6.

### Statistical analysis

GraphPad Prism 8 was used for all statistical analysis. All data sets were generated with at least three replicates unless specified otherwise, and error bars are reported as mean ± SD. Ordinary one-way ANOVA Dunnett’s tests were used to measure significance of data of multiple groups, and unpaired t-tests were used to measure significance of data of two groups with confidence level cutoff of ns, not significant ≥0.05, *p < 0.05 **p < 0.01, ***p < 0.001, ****p < 0.0001.

### Reporting summary

Further information on research design is available in the Nature Portfolio Reporting Summary linked to this article.

### Supplementary information

Supplementary Information

Reporting Summary

### Source data

Source Data

## Data Availability

The key plasmids generated from this study have been deposited on Addgene (http://www.addgene.org). Yeast strains used in this study are available upon request to the corresponding authors. Source data are provided in this paper.
