# Supplementary material for: Enhanced eMAGE applied to identify genetic factors of nuclear hormone receptor dysfunction via combinatorial gene editing
Source: Nat Commun. 2024 Jun 18;15:5218. doi: 10.1038/s41467-024-49365-z (PMC11189492; doi:10.1038/s41467-024-49365-z)
Supplement: Supplementary file 1 — Supplementary Information [file 41467_2024_49365_MOESM1_ESM.pdf]

Enhanced eMAGE applied to identify genetic factors of nuclear hormone receptor dysfunction via combinatorial gene editing

Peter N. Ciaccia[1,2,3*], Zhuobin Liang[1,2,5*+], Anabel Y. Schweitzer[1,2], Eli Metzner[1,2], and Farren J. Isaacs[1,2,4,+]

[1]Department of Molecular, Cellular, and Developmental Biology, Yale University, New Haven, CT 06520, USA
[2]Systems Biology Institute, Yale University, West Haven, CT 06516, USA
[3]Physical and Engineering Biology, Yale University, New Haven, CT 06520, USA
[4]Department of Biomedical Engineering, Yale University, New Haven, CT 06520, USA
[5]Present address: ZL: Institute of Molecular Physiology, Shenzhen Bay Laboratory, Shenzhen 518132, China.
* These authors contributed equally: Peter N. Ciaccia, Zhuobin Liang
[+]Lead Contact/Correspondence addressed to: FJI (farren.isaacs@yale.edu) and ZL (zbliang@szbl.ac.cn)

Supplementary Information

**Supplementary Table 1:**	**Subunit variants of DNA mismatch repair MutS complexes q tested in this study**
**Supplementary Table 2:**	**Influence of MMR proficiency on eMAGE genome editing and spontaneous mutations**
**Supplementary Table 3:**	**Yeast strains used in this study**
**Supplementary Table 4:**	**Plasmids used in this study**
**Supplementary Table 5:**	**ssODNs used in this study excluding the dual hormone biosensor**
**Supplementary Table 6:**	**Targeted substitutions and ssODNs used for the dual hormone biosensor**
**Supplementary Figure 1:**	**Effect of ssODN concentration and modification on frequency of edited cells and cell viability**
**Supplementary Figure 2:**	**Comparison of dual marker co-selection using ADE2 vs TRP1 as a second marker**
**Supplementary Figure 3:**	**Comparison of three dual-marker placement strategies**
**Supplementary Figure 4:**	**MMR deficiency promotes eMAGE editing and benefits implementation of dual-marker co-selection**
**Supplementary Figure 5:**	**Engineering tunable expression of dominant negative mutants to modulate MMR fidelity during eMAGE editing**
**Supplementary File 1:**	**DNA sequence files of important genomic loci and plasmids**

Supplementary Table 1: Subunit variants of DNA mismatch repair MutS complexes tested in this study.

MMR gene	Variant	Mutated domain	Protein biochemistry	Reported spontaneous mutation rate[a] (vs. wild-type)	Sources
**MSH2**	Wild-type	-	-	Unchanged[b]	Alani et al, *Mol Cell Biol.* 1997[26]
	G693D	ATPase (MSH2)	Defective ATP binding	~50-fold[b]	
	G855D		Defective ATP binding, hydrolysis and MutS-DNA complex formation		
	G693D, G855D (GGDD)		Combined effects of two single mutants		
**MSH6**	Wild-type	-	-	Unchanged[b]	Gupta et al, *Nat Genet.* 2000[28]
	S1036P	ATPase (MSH6)	Defective ATP hydrolysis	~15-fold[b]	
	G1067D	Domain interacting with ATPase (MSH2)	Defective ATP hydrolysis and MutS-DNA complex formation		
	G1142D				
**MLH1**	Wild-type	-	Imbalanced MLH1-PSM1 stoichiometry	~13-fold[b]	Shcherbakova et al, *Mol Cell Biol.* 1999[27]
	G64R	ATPase (MLH1)	Defective ATP binding and hydrolysis	~6-fold[c]	
	I65N				
	T114M				

[a] Based on reported data of the referenced literatures, showing the fold increases of mutation rate upon overexpression of specific MMR proteins as compared to the corresponding wild-type strains.

[b] Overexpressed from high-copy 2μ plasmids. Mutation rates of *CAN1* gene were measured by quantification of canavanine resistance colonies.

[c] Expressed from the endogenous *MLH1* locus. Mutation rates of *lys2*::*InsE-A14* locus were measured by quantification of Lys^+ colonies.

Supplementary Table 2: Influence of MMR proficiency on eMAGE genome editing and spontaneous mutations.

	**A**	**B**		**C**	**D**	**E**
**MMR status**	**Spontaneous mutation rate** (*ura3* per cell per division)	**Est. number of spontaneous mutations** (per cell)		**Avg. number of on-target edits** (per cell per eMAGE)	**Approx. number of URA3, ADE2 cells** (electrop. cells: 10^9)	**Total number of eMAGE edited sites** (edited sites=**C**×**D**)
		eMAGE[a]	10-day cultivation[b]			
Wild-type	$\mathbf{5.45\times10^{-8}}$	0.05	0.10	1.4	$\sim1.5\times10^3$	2.1×10^3
*msh2*Δ	$\mathbf{1.38\times10^{-6}}$	1.37	2.50	1.7	$\sim1.5\times10^4$	2.6×10^4
Wild-type + p[MSH2-GGDD]	$\mathbf{8.02\times10^{-8}}$ (uninduced)<br>$\mathbf{1.93\times10^{-6}}$ (β-E: 0.6 μM)	0.33	0.15	1.7	$\sim1.5\times10^4$	2.6×10^4

[a] One round of eMAGE involves ~66 cell divisions; MSH2-GGDD was induced for 18 hours (~9 cell divisions). Detailed calculations in Method.

[b] Est. cultivation time of eMAGE relevant applications (*e.g.*, metabolic variant selection or engineered protein production).

Note: Column **A** shows spontaneous mutation rate of yeast strains determined by fluctuation analysis of *URA3* loss-of-function mutagenesis. The MMR fidelity of the strain carrying p[MSH2-GGDD] (episomal vector of MSH2-GGDD inducible expression) can be toggled between the levels of wild-type (uninduced) and *msh2*Δ (β-E: 0.6 μM). Mutation rates of MMR wild-type and *msh2*Δ strains observed in our study are consistent with previous reports[53]. Column **B** shows the estimated number of spontaneous mutations in the compared strains. Because MSH2-GGDD is only expressed prior to ssODN electroporation for 18 hours out of the 5.5 days of total cultivation time needed for one round of eMAGE (2-day of isolated colony forming from frozen glycerol stock and 3.5-day of eMAGE protocol time), the transient expression scheme greatly reduces the accumulated spontaneous mutations during one round of eMAGE by >4-fold as compared to *msh2*Δ cells, to a level similar to the MMR-proficient (wild-type) cells (column **B**, eMAGE). Notably, eMAGE strains often go through multiple rounds of editing and are used in applications that require prolonged cultivation. Therefore, wild-type levels of genome stability of the eMAGE strain carrying p[MSH2-GGDD] lead to a substantial ~17-fold reduction of secondary mutations during cell growth (column **B**, 10-day cultivation). Meanwhile, tunable expression of MSH2-GGDD allows *msh2*Δ levels of high editing efficiency (column **C**) and provides a 10-fold increase of double-edited URA3, ADE2 cells (column **D**), resulting in a 10-fold higher diversification capacity for generating up to 10^4 unique variants as compared to MMR wild-type strain (column **E**). β-E: β-estradiol. Data sources and calculations involved in this table can be found in Method.

Supplementary Table 3. Yeast strains used in this study

Name	Genotype	Episomal vector	Ancestor	Associated figures or tables	Description	Source	Sequence map
**SZL114**	*MAT**a** his3Δ1 leu2Δ0 met15Δ0 ura3Δ0 ARS1516-URA3-ADE2*	-	**BY4741**	-	*URA3-ADE2* reporter (MMR-WT)	Barbieri et al, *Cell* 2017[11]	Upon request
**SZL149**	*MAT**a** his3Δ1 leu2Δ0 met15Δ0 ura3Δ0 ARS1516-ymUkG1(FS)-yEmRFP(FS)-ADE2*	-	**SZL114**	-	*GFP*(FS)-*RFP*(FS) reporter (MMR-WT)	This Study	Upon request
**SZL238**	*MAT**a** his3Δ1 leu2Δ0 met15Δ0 ura3Δ0 ARS1516-URA3-yEmRFP(FS)-ADE2*	-	**SZL149**	-	*URA3-RFP*(FS) reporter (MMR-WT)	This Study	Upon request
**SZL247**	*MAT**a** his3Δ1 leu2Δ0 met15Δ0 ura3Δ0 ARS1516-URA3(FS)-yEmRFP(FS)-ADE2*	-	**SZL238**	-	*URA3*(FS)-*RFP*(FS) reporter (MMR-WT)	This Study	Upon request
**SZL335**	*MAT**a** his3Δ1 leu2Δ0 met15Δ0 ura3Δ0 ARS1516-URA3(FS)-yEmRFP(FS)-ADE2 msh2Δ::KanMX4*	-	**SZL247**	Fig. 1e; Supplementary Fig. 1a-d	*URA3*(FS)-*RFP*(FS) reporter (*msh2*Δ)	This Study	**Supplementary File 1**
**SZL207**	*MAT**a** his3Δ1 leu2Δ0 met15Δ0 ura3Δ0 ARS1516-ymUkG1(FS)-yEmRFP(FS)-ADE2 msh2Δ::KanMX4*	-	**SZL149**	-	*GFP*(FS)-*RFP*(FS)) reporter (*msh2*Δ), no GEM exp.	This Study	Upon request
**SZL240**	*MAT**a** his3Δ1 leu2Δ0 met15Δ0 ura3Δ0 YFL033C-LEU2-GEM ARS1516-URA3-ADE2*	-	**SZL114**	-	*URA3-ADE2* reporter (MMR-WT), with GEM exp.	This Study	Upon request
**SZL281**	*MAT**a** his3Δ1 leu2Δ0 met15Δ0 ura3Δ0 YFL033C-LEU2-GEM ARS1516-ymUkG1(FS)-yEmRFP(FS)-ADE2*	-	**SZL149**	-	*GFP*(FS)-*RFP*(FS) reporter (MMR-WT strain)	This Study	Upon request
**SZL308**	*MAT**a** his3Δ1 leu2Δ0 met15Δ0 ura3Δ0 YFL033C-LEU2-GEM ARS1516-ymUkG1(FS)-yEmRFP(FS)-ADE2 msh2Δ::KanMX4*	-	**SZL207**	-	*GFP*(FS)-*RFP*(FS)) reporter (*msh2*Δ)	This Study	Upon request
**SZL345**	*MAT**a** his3Δ1 leu2Δ0 met15Δ0 ura3Δ0 YFL033C-LEU2-GEM ARS1516-URA3-ymTaqBFP2(FS)-ymUkG1(FS)-yEmRFP(FS)-ADE2*	-	**SZL281**	Supplementary Table 2	*URA3-BGR*(FS)-*ADE2*(FS) reporter (MMR-WT strain)	This Study	Upon request
**SZL347**	*MAT**a** his3Δ1 leu2Δ0 met15Δ0 ura3Δ0 YFL033C-LEU2-GEM ARS1516-URA3-ymTaqBFP2(FS)-ymUkG1(FS)-yEmRFP(FS)-ADE2 msh2Δ::KanMX4*	-	**SZL308**	Supplementary Fig. 2; Supplementary Table 2	*URA3-BGR*(FS)-*ADE2*(FS) reporter (*msh2*Δ)	This Study	Upon request
**SZL376**	*MAT**a** his3Δ1 leu2Δ0 met15Δ0 ura3Δ0 YFL033C-LEU2-GEM ARS1516-URA3(FS)-ymTaqBFP2(FS)-ymUkG1(FS)-yEmRFP(FS)-ADE2(FS)*	-	**SZL345**	Fig. 3b; Supplementary Fig. 3; 4c,d	*URA3*(FS)-*BGR*(FS)-*ADE2*(FS) reporter (MMR-WT strain)	This Study	**Supplementary File 1**
**SZL348**	*MAT**a** his3Δ1 leu2Δ0 met15Δ0 ura3Δ0 YFL033C-LEU2-GEM ARS1516-URA3(FS)-ymTaqBFP2(FS)-ymUkG1(FS)-yEmRFP(FS)-ADE2(FS) msh2Δ::KanMX4*	-	**SZL347**	Fig. 2b,c; Fig. 3b; Supplementary Fig. 3; 4c,d	*URA3*(FS)-*BGR*(FS)-*ADE2*(FS) reporter (*msh2*Δ)	This Study	**Supplementary File 1**
**SZL241**	*MAT**a** his3Δ1 leu2Δ0 met15Δ0 ura3Δ0 YFL033C-LEU2-GEM ARS1516-URA3-ADE2*	**SZL231**	**SZL240**	Supplementary Fig. 4a	Episomal overexpression of yEmRFP	This Study	**Supplementary File 1**
**SZL360**	*MAT**a** his3Δ1 leu2Δ0 met15Δ0 ura3Δ0 YFL033C-LEU2-GEM ARS1516-URA3(FS)-ymTaqBFP2(FS)-ymUkG1(FS)-yEmRFP(FS)-ADE2(FS)*	**SZL369**	**SZL345**	Supplementary Table 2	Fluctuation analysis of mutation rate with or w/o MSH2-GGDD exp.	This Study	**Supplementary File 1**
**SZL432**	*MAT**a** his3Δ1 leu2Δ0 met15Δ0 ura3Δ0 YFL033C-LEU2-GEM ARS1516-URA3(FS)-ymTaqBFP2(FS)-ymUkG1(FS)-yEmRFP(FS)-ADE2(FS)*	**SZL369**	**SZL376**	Fig. 3b; Supplementary Fig. 4c,d	Episomal overexpression of MSH2-GGDD	This Study	**Supplementary File 1**
**SZL433**	*MAT**a** his3Δ1 leu2Δ0 met15Δ0 ura3Δ0 YFL033C-LEU2-GEM ARS1516-URA3(FS)-ymTaqBFP2(FS)-ymUkG1(FS)-yEmRFP(FS)-ADE2(FS)*	**SZL370**	**SZL376**	Fig. 3b; Supplementary Fig. 4c,d	Episomal overexpression of MLH1-WT	This Study	**Supplementary File 1**
**SZL434**	*MAT**a** his3Δ1 leu2Δ0 met15Δ0 ura3Δ0 YFL033C-LEU2-GEM ARS1516-URA3(FS)-ymTaqBFP2(FS)-ymUkG1(FS)-yEmRFP(FS)-ADE2(FS)*	**SZL410**	**SZL376**	Fig. 3b; Supplementary Fig. 4d	Episomal overexpression of both MSH2-GGDD & MLH1-WT	This Study	**Supplementary File 1**
**SZL486**	*MAT**a** his3Δ1 leu2Δ0 met15Δ0 ura3Δ0 YFL033C-LEU2-GEM ARS1516-URA3(FS)-ymTaqBFP2(FS)-ymUkG1(FS)-yEmRFP(FS)-ADE2(FS)*	**SZL144**	**SZL377**	Supplementary Fig. 4c	Episomal overexpression of MSH6-WT	This Study	**Supplementary File 1**
**SZL487**	*MAT**a** his3Δ1 leu2Δ0 met15Δ0 ura3Δ0 YFL033C-LEU2-GEM ARS1516-URA3(FS)-ymTaqBFP2(FS)-ymUkG1(FS)-yEmRFP(FS)-ADE2(FS)*	**SZL145**	**SZL378**	Supplementary Fig. 4c	Episomal overexpression of MSH6-G1142D	This Study	Upon request
**SZL488**	*MAT**a** his3Δ1 leu2Δ0 met15Δ0 ura3Δ0 YFL033C-LEU2-GEM ARS1516-URA3(FS)-ymTaqBFP2(FS)-ymUkG1(FS)-yEmRFP(FS)-ADE2(FS)*	**SZL364**	**SZL379**	Supplementary Fig. 4c	Episomal overexpression of MSH6-G1067D	This Study	Upon request
**SZL489**	*MAT**a** his3Δ1 leu2Δ0 met15Δ0 ura3Δ0 YFL033C-LEU2-GEM ARS1516-URA3(FS)-ymTaqBFP2(FS)-ymUkG1(FS)-yEmRFP(FS)-ADE2(FS)*	**SZL365**	**SZL380**	Supplementary Fig. 4c	Episomal overexpression of MSH6-S1036P	This Study	Upon request
**SZL490**	*MAT**a** his3Δ1 leu2Δ0 met15Δ0 ura3Δ0 YFL033C-LEU2-GEM ARS1516-URA3(FS)-ymTaqBFP2(FS)-ymUkG1(FS)-yEmRFP(FS)-ADE2(FS)*	**SZL366**	**SZL381**	Supplementary Fig. 4c	Episomal overexpression of MSH2-WT	This Study	**Supplementary File 1**
**SZL491**	*MAT**a** his3Δ1 leu2Δ0 met15Δ0 ura3Δ0 YFL033C-LEU2-GEM ARS1516-URA3(FS)-ymTaqBFP2(FS)-ymUkG1(FS)-yEmRFP(FS)-ADE2(FS)*	**SZL367**	**SZL382**	Supplementary Fig. 4c	Episomal overexpression of MSH2-G693D	This Study	Upon request
**SZL492**	*MAT**a** his3Δ1 leu2Δ0 met15Δ0 ura3Δ0 YFL033C-LEU2-GEM ARS1516-URA3(FS)-ymTaqBFP2(FS)-ymUkG1(FS)-yEmRFP(FS)-ADE2(FS)*	**SZL368**	**SZL383**	Supplementary Fig. 4c	Episomal overexpression of MSH2-G855D	This Study	Upon request
**SZL493**	*MAT**a** his3Δ1 leu2Δ0 met15Δ0 ura3Δ0 YFL033C-LEU2-GEM ARS1516-URA3(FS)-ymTaqBFP2(FS)-ymUkG1(FS)-yEmRFP(FS)-ADE2(FS)*	**SZL371**	**SZL384**	Supplementary Fig. 4c	Episomal overexpression of MLH1-G64R	This Study	Upon request
**SZL494**	*MAT**a** his3Δ1 leu2Δ0 met15Δ0 ura3Δ0 YFL033C-LEU2-GEM ARS1516-URA3(FS)-ymTaqBFP2(FS)-ymUkG1(FS)-yEmRFP(FS)-ADE2(FS)*	**SZL372**	**SZL385**	Supplementary Figs. 4c	Episomal overexpression of MLH1-I65N	This Study	Upon request
**SZL495**	*MAT**a** his3Δ1 leu2Δ0 met15Δ0 ura3Δ0 YFL033C-LEU2-GEM ARS1516-URA3(FS)-ymTaqBFP2(FS)-ymUkG1(FS)-yEmRFP(FS)-ADE2(FS)*	**SZL373**	**SZL386**	Supplementary Fig. 4c	Episomal overexpression of MLH1-T114M	This Study	Upon request
**PCY374**	*MATa his3Δ1 leu2Δ0 met15Δ0 ura3Δ0 msh2Δ::KanMX4 ARS1516-URA3-DHB-TRP1(FS)-ADE2(FS)*	-	**SZL335**	-	Dual hormone biosensor	This Study	Upon request
**PCY421**	*MATa his3Δ1 leu2Δ0 met15Δ0 ura3Δ0 msh2Δ::KanMX4 ARS1516-URA3(FS)-DHB-TRP1(FS)-ADE2(FS)*	-	**PCY374**	Fig. 4	Dual hormone biosensor	This Study	**Supplementary File 1**

Supplementary Table 4. Plasmids used in this study

Name	Episomal vector	*E. coli* strain	Associated Figures	Description	Source
**SZL143**	pRSII42B	DH10B	-	The LEU2 cassette of pRSII425 (Addgene #35468) was replaced by a *ble* cassette conferring zeocin resistance	This study
**SZL144**	pRSII42B[Gal1p-MSH6]	DH10B	Ext. Data Fig. 4c	Vector for β-estradiol (or galactose) inducible expression of MSH6-WT	This study
**SZL145**	pRSII42B[Gal1p-MSH6(G1142D)]	DH10B	Ext. Data Fig. 4c	Vector for β-estradiol (or galactose) inducible expression of MSH6-G1142D	This study
**SZL364**	pRSII42B[Gal1p-MSH6(G1067D)]	DH10B	Ext. Data Fig. 4c	Vector for β-estradiol (or galactose) inducible expression of MSH6-G1067D	This study
**SZL365**	pRSII42B[Gal1p-MSH6(S1036P)]	DH10B	Ext. Data Fig. 4c	Vector for β-estradiol (or galactose) inducible expression of MSH6-S1036P	This study
**SZL366**	pRSII42B[Gal1p-MSH2]	DH10B	Ext. Data Fig. 4c	Vector for β-estradiol (or galactose) inducible expression of MSH2-WT	This study
**SZL367**	pRSII42B[Gal1p-MSH2(G693D)]	DH10B	Ext. Data Fig. 4c	Vector for β-estradiol (or galactose) inducible expression of MSH2-G693D	This study
**SZL368**	pRSII42B[Gal1p-MSH2(G855D)]	DH10B	Ext. Data Fig. 4c	Vector for β-estradiol (or galactose) inducible expression of MSH2-G855D	This study
**SZL369**	pRSII42B[Gal1p-MSH2(G693D, G855D)]	DH10B	Fig. 3b; Ext. Data Fig. 3c,d	Vector for β-estradiol (or galactose) inducible expression of MSH2-GGDD	This study
**SZL370**	pRSII42B[Gal1p-MLH1]	DH10B	Fig. 3b; Ext. Data Fig. 3c,d	Vector for β-estradiol (or galactose) inducible expression of MLH1-WT	This study
**SZL371**	pRSII42B[Gal1p-MLH1(G64R)]	DH10B	Ext. Data Fig. 4c	Vector for β-estradiol (or galactose) inducible expression of MLH1-G64R	This study
**SZL372**	pRSII42B[Gal1p-MLH1(I65N)]	DH10B	Ext. Data Fig. 4c	Vector for β-estradiol (or galactose) inducible expression of MLH1-I65N	This study
**SZL373**	pRSII42B[Gal1p-MLH1(T114M)]	DH10B	Ext. Data Fig. 4c	Vector for β-estradiol (or galactose) inducible expression of MLH1-T114M	This study
**SZL410**	pRSII425[Gal1p-MSH2(GGDD)_Ga10p-MLH1]	DH10B	Fig. 3b; Ext. Data Fig. 4d	Vector for β-estradiol (or galactose) inducible co-expression of MSH2-GGDD and MLH1-WT	This study
**SZL231**	pRSII41B[Gal1p-yEmRFP]	DH10B	Ext. Data Fig. 3a	Vector for β-estradiol (or galactose) inducible expression of yEmRFP	This study

Supplementary Table 5. ssODNs used in this study excluding the dual hormone biosensor

Name	Sequence (5'-3')	Modification	Application	Associated figures
**OZL115**	AATTGAACAACCTTCTTGGCCTTGTAAGAAGTCTTGAAATCACATCTATG<br>ATGACCACCACCTTCCAACAACAAAGCCATATTGACATCA	N/A	eMAGE ssODN to correct *GFP*(FS), and non-targeting ssODN in assays of Fig. 1e	Fig. 1e; 2b,c; 3b; Supplementary Fig. 2b; 3a; 4c,d
**OZL116**	CCTTCACCTTCACCTTCAATTTCAAATTCATGACCATTAACTGAACCTTC<br>CATATGAACTTTAAATCTCATAAATTCTTTAATAATAGCC	N/A	eMAGE ssODN to correct *RFP*(FS)	Fig. 1e; 2b,c; 3b; Supplementary Fig. 1a-d; 2b; 3a; 4c,d
**OZL326**	CGTGGATGATGTGGTCTCTACAGGATCTGACATTATTATTGTTGGAAGAG<br>GACTATTTGCAAAGGGAAGGGATGCTAAGGTAGAGGGTGA	N/A	eMAGE ssODN to correct *URA3*(FS)	Fig. 1e; 2b,c; 3b; Supplementary Fig. 1a-d; 3a; 4c,d
**OZL327**	CGTGGATGATGTGGTCTCTACAGGATCTGACATTATTATTGTTGtGAAGA<br>GGACTATTTGCAAAGGGAAGGGATGCTAAGGTAGAGGGTG	N/A	eMAGE ssODN to introduce *URA3*(FS)	-
**OZL427**	ACCACCTTCAACAACCTTGATTCTCATGGTTTGAGTACCTTCATATGGTT<br>TACCTTCACCTTCAGAGGTACACTTGAAATGATGGTTATC	N/A	eMAGE ssODN to correct *BFP*(FS)	Fig. 2b,c; 3b Supplementary Fig. 2b; 3a; 4c,d
**OZL428**	ACGTGGTCATTGGAGTTGCTTATTTGTTTGGCAGGAGAATTTTCaAGCAT<br>CTAGTATTACCGTCTTAATGTTGAGCCTGTTTGCTGCCTC	N/A	eMAGE ssODN to correct *ADE2*(FS)	Fig. 2b,c; 3b; Supplementary Fig. 3a; 4c,d
**OZL429**	AACGTGGTCATTGGAGTTGCTTATTTGTTTGGCAGGAGAATTTTCAGCAT<br>CTAGTATTACCGTCTTAATGTTGAGCCTGTTTGCTGCCTC	N/A	eMAGE ssODN to introduce *ADE2*(FS)	-
**OZL472**	C*G*T*G*GATGATGTGGTCTCTACAGGATCTGACATTATTATTGTTGGA<br>AGAGGACTATTTGCAAAGGGAAGGGATGCTAAGGTAGAGG*G*T*G*A	*phosphorothioate	eMAGE ssODN to correct *URA3*(FS)	Supplementary Fig. 1d
**OZL473**	C*C*T*T*CACCTTCACCTTCAATTTCAAATTCATGACCATTAACTGAAC<br>CTTCCATATGAACTTTAAATCTCATAAATTCTTTAATAAT*A*G*C*C	*phosphorothioate	eMAGE ssODN to correct *RFP*(FS)	Supplementary Fig. 1d
**BDL4001**	AGAAGCAGGTGGGACAGGTGAACTTTTGGATTGGAACTCGATTTCTGACT<br>GGGTTGGAAGGCAAGAGAGCCCCGAAAGCTTACATTTTAT	N/A	eMAGE ssODN to correct *TRP1* (FS)	Fig. 2d, 4

Supplementary Table 6. Targeted substitutions and ssODNs used for the dual hormone biosensor

Gene	Target	ssODN	Sequence	ΔG
ESR1	S329	oST_GEM_S209X-T4263N_C4264N_C4265K	tcatcgaagcttcactgaagggtctggtaggatcatactcMNNatagagtatgggggctcagcatccaacaaggcactgaccatctggt	-7.2
ESR1	M343	oST_GEM_M223X-A4305N_T4306N_G4307K	accctcttcgcccagttgatcatgtgaaccagctccctgtctgccaggttggtcagtaagccMNNcatcgaagcttcactgaagggtctg	-9
ESR1	G344	oST_GEM_G224X-G4308N_G4309N_C4310K	ccctcttcgcccagttgatcatgtgaaccagctccctgtctgccaggttggtcagtaaMNNcatcatcgaagcttcactgaagggtctgg	-6.6
ESR1	T347	oST_GEM_T227X-A4317N_C4318N_C4319K	accctcttcgcccagttgatcatgtgaaccagctccctgtctgccaggttMNNcagtaagcccatcatcgaagcttcactgaagggtctg	-5.7
ESR1	L349	oST_GEM_L229X-C4323N_T4324N_G4325K	tccacaaagcctggcaccctcttcgcccagttgatcatgtgaaccagctccctgtctgcMNNgttggtcagtaagcccatcatcgaagct	-3
ESR1	A350	oST_GEM_A230X-G4326N_C4327N_A4328K	atccacaaagcctggcaccctcttcgcccagttgatcatgtgaaccagctccctgtcMNNcaggttggtcagtaagcccatcatcgaagc	-4.8
ESR1	D351	oST_GEM_D231X-G4329N_A4330N_C4331K	atccacaaagcctggcaccctcttcgcccagttgatcatgtgaaccagctccctMNNtgccaggttggtcagtaagcccatcatcgaagc	-6.2
ESR1	E353	oST_GEM_E233X-G4335N_A4336N_G4337K	atccacaaagcctggcaccctcttcgcccagttgatcatgtgaaccagMNNcctgtctgccaggttggtcagtaagcccatcatcgaagc	-6.9
ESR1	L354	oST_GEM_L234X-C4338N_T4339N_G4340K	atccacaaagcctggcaccctcttcgcccagttgatcatgtgaacMNNctccctgtctgccaggttggtcagtaagcccatcatcgaagc	-5.59
ESR1	E380	oST_GEM_E260X-G4416N_A4417N_A4418K	gagcgccagacgagaccaatcatcaggatctctagccaggcacaMNNtagaaggtggacctgatcatggagggtcaaatccacaaagcct	-6.2
ESR1	E380	oST_GEM_E260Q-G4416C	gagcgccagacgagaccaatcatcaggatctctagccaggcacattGtagaaggtggacctgatcatggagggtcaaatccacaaagcct	-7.4
ESR1	L387	oST_GEM_L267X-C4437N_T4438N_G4439K	gagcgccagacgagaccaatcatMNNgatctctagccaggcacattctagaaggtggacctgatcatggagggtcaaatccacaaagcct	-6.2
ESR1	M388	oST_GEM_M268X-A4440N_T4441N_G4442K	gagcgccagacgagaccaatMNNcaggatctctagccaggcacattctagaaggtggacctgatcatggagggtcaaatccacaaagcct	-6.6
ESR1	R394	oST_GEM_R274X-C4458N_G4459N_C4460K	caaacagtagcttccctgggtgctccatggaMNNccagacgagaccaatcatcaggatctctagccaggcacattctagaaggtggacct	-6.6
ESR1	R394	oST_GEM_R274X-C4458N_G4459N_C4460K	caaacagtagcttccctgggtgctccatggaMNNccagacgagaccaatcatcaggatctctagccaggcacattctagaaggtggacct	-6.6
ESR1	G400	oST_GEM_G280V-G4477T	ctctacacattttccctggttcctgtccaagagcaagttaggagcaaacagtagcttcActgggtgctccatggagcgccagacgagacc	-9.6
ESR1	F404	oST_GEM_F284X-T4488N_T4489N_T4490K	aagatctccaccatgccctctacacattttccctggttcctgtccaagagcaagttaggagcMNNcagtagcttccctgggtgctccatg	-4.4
ESR1	N407	oST_GEM_N287X-A4497N_A4498N_C4499K	catgtcgaagatctccaccatgccctctacacattttccctggttcctgtccaagagcaaMNNaggagcaaacagtagcttccctgggtg	-3.2
ESR1	C417	oST_GEM_C297X-T4527N_G4528N_T4529K	catgtcgaagatctccaccatgccctctacMNNttttccctggttcctgtccaagagcaagttaggagcaaacagtagcttccctgggtg	-3.7
ESR1	V418	oST_GEM_V298X-G4530N_T4531N_A4532K	catgtcgaagatctccaccatgccctcMNNacattttccctggttcctgtccaagagcaagttaggagcaaacagtagcttccctgggtg	-2.9
ESR1	M421	oST_GEM_M301X-A4539N_T4540N_G4541K	cagcagcatgtcgaagatctccacMNNgccctctacacattttccctggttcctgtccaagagcaagttaggagcaaacagtagcttccc	-1.9
ESR1	S432	oST_GEM_S312X-T4572N_C4573N_A4574K	gatttgaggcacacaaactcctctccctgcagattcatcatgcggaaccgagaMNNtgtagccagcagcatgtcgaagatctccaccatg	-4.5
ESR1	K449	oST_GEM_K329X-A4623N_A4624N_A4625K	acagaaatgtgtacactccagaattaagcaaaataatagaMNNgaggcacacaaactcctctccctgcagattcatcatgcggaaccgag	-2.8
ESR1	K449	oST_GEM_K329Q-A4623C	actccagaattaagcaaaataatagattGgaggcacacaaactcctctccctgcagattcatcatgcggaaccgagatgatgtagccagc	-6.6
ESR1	F461	oST_GEM_F341X-T4659N_T4660N_T4661K	cttccagagacttcagggtgctggacagMNNtgtgtacactccagaattaagcaaaataatagatttgaggcacacaaactcctctccct	-5.6
ESR1	S463	oST_GEM_S343X-T4665N_C4666N_C4667K	tcttccagagacttcagggtgctMNNcagaaatgtgtacactccagaattaagcaaaataatagatttgaggcacacaaactcctctccc	-3.2
ESR1	L466	oST_GEM_L346X-C4674N_T4675N_G4676K	gactcggtggatatggtccttctcttccagagacttMNNggtgctggacagaaatgtgtacactccagaattaagcaaaataatagattt	-5.8
ESR1	L469	oST_GEM_L349X-C4683N_T4684N_G4685K	gactcggtggatatggtccttctcttcMNNagacttcagggtgctggacagaaatgtgtacactccagaattaagcaaaataatagattt	-4.6
ESR1	V478	oST_GEM_V358X-G4710N_T4711N_C4712K	gtgtctgtgatcttgtccagMNNtcggtggatatggtccttctcttccagagacttcagggtgctggacagaaatgtgtacactccagaa	-6.8
ESR1	M522	oST_GEM_M402X-A4842N_T4843N_G4844K	ggggcaccacgttcttgcacttcatgctgtacagatgctcMNNgcctttgttactcatgtgcctgatgtgggagaggatgaggaggagct	-5.4
ESR1	H524	oST_GEM_H404X-C4848N_A4849N_T4850K	ggtcatagaggggcaccacgttcttgcacttcatgctgtacagMNNctccatgcctttgttactcatgtgcctgatgtgggagaggatga	-4.6
ESR1	N532	oST_GEM_N412X-A4872N_A4873N_C4874K	aggtcatagaggggcaccacMNNcttgcacttcatgctgtacagatgctccatgcctttgttactcatgtgcctgatgtgggagaggatg	-5.1
ESR1	V533	oST_GEM_V413X-G4875N_T4876N_G4877K	agcaggtcatagaggggcacMNNgttcttgcacttcatgctgtacagatgctccatgcctttgttactcatgtgcctgatgtgggagagg	-6
ESR1	V534	oST_GEM_V414X-G4878N_T4879N_G4880K	agcagcaggtcatagaggggMNNcacgttcttgcacttcatgctgtacagatgctccatgcctttgttactcatgtgcctgatgtgggag	-6.1
ESR1	P535	oST_GEM_P415X-C4881N_C4882N_C4883K	ctccagcagcaggtcatagagMNNcaccacgttcttgcacttcatgctgtacagatgctccatgcctttgttactcatgtgcctgatgtg	-5.6
ESR1	L536	oST_GEM_L416X-C4884N_T4885N_C4886K	catctccagcagcaggtcataMNNgggcaccacgttcttgcacttcatgctgtacagatgctccatgcctttgttactcatgtgcctgat	-5.4
ESR1	Y537	oST_GEM_Y417X-T4887N_A4888N_T4889K	cctccacggctagtgggcgcatgtaggcggtgggcgtccagcatctccagcagcaggtcMNNgaggggcaccacgttcttgcacttcatg	-8.7
ESR1	Y537	oST_GEM_Y417C-A4888G	cctccacggctagtgggcgcatgtaggcggtgggcgtccagcatctccagcagcaggtcaCagaggggcaccacgttcttgcacttcatg	-9
ESR1	L539	oST_GEM_L419A_L420A-C4893G_T4894C_C4896G_T4897C	gggcgtccagcatctccagcGCcGCgtcatagaggggcaccacgttcttgcacttcatgctgtacagatgctccatgcctttgttactca	-10.1
ESR1	L540	oST_GEM_L420X-C4896N_T4897N_G4898K	ccctccacggctagtgggcgcatgtaggcggtgggcgtccagcatctccagMNNcaggtcatagaggggcaccacgttcttgcacttcat	-8.3
ESR1	L540	oST_GEM_L420Q-T4897A	agtgggcgcatgtaggcggtgggcgtccagcatctccagcTgcaggtcatagaggggcaccacgttcttgcacttcatgctgtacagatg	-10.1
ESR1	L541	oST_GEM_L421X-C4899N_T4900N_G4901K	tagtgggcgcatgtaggcggtgggcgtccagcatctcMNNcagcaggtcatagaggggcaccacgttcttgcacttcatgctgtacagat	-8.5
ESR1	E542	oST_GEM_E422X-G4902N_A4903N_G4904K	catgtaggcggtgggcgtccagcatMNNcagcagcaggtcatagaggggcaccacgttcttgcacttcatgctgtacagatgctccatgc	-8.1
ESR1	M543	oST_GEM_M423A_L424A-A4905G_T4906C_C4908G_T4909C	cgcatgtaggcggtgggcgtccGCcGCctccagcagcaggtcatagaggggcaccacgttcttgcacttcatgctgtacagatgctccat	-15.5
ESR1	A546	oST_GEM_A426X-G4914N_C4915N_C4916K	tagtgggcgcatgtaggcggtgMNNgtccagcatctccagcagcaggtcatagaggggcaccacgttcttgcacttcatgctgtacagat	-7.6
ESR1	L549	oST_GEM_L429X-C4923N_T4924N_A4925K	cctccacggctagtgggcgcatgMNNgcggtgggcgtccagcatctccagcagcaggtcatagaggggcaccacgttcttgcacttcatg	-8.7
PGR	L715	oST_ZPM_L171X-C10130N_T10131N_G10132K	aaagaagttgcctctcgcctagttgattaagacttgtMNNcaaagaactggaggtgtcaggttttgtgttgtcatgtcctgcatagatca	-3.2
PGR	L718	oST_ZPM_L174X-C10139N_T10140N_T10141K	aaagaagttgcctctcgcctagttgattMNNacttgtcagcaaagaactggaggtgtcaggttttgtgttgtcatgtcctgcatagatca	-3.2
PGR	N719	oST_ZPM_N175X-A10142N_A10143N_T10144K	gaaaacctggcaatgatttagaccacttgactactgaaagaagttgcctctcgcctagttgMNNaagacttgtcagcaaagaactggagg	-2.4
PGR	L721	oST_ZPM_L177X-C10148N_T10149N_A10150K	tttcgaaaacctggcaatgatttagaccacttgactactgaaagaagttgcctctcgccMNNttgattaagacttgtcagcaaagaactg	-2.3
PGR	Q725	oST_ZPM_Q181X-C10160N_A10161N_A10162K	tttcgaaaacctggcaatgatttagaccacttgactactgaaagaagMNNcctctcgcctagttgattaagacttgtcagcaaagaactg	-0.5
PGR	R740	oST_ZPM_R196X-C10205N_G10206N_A10207K	ccaagaatactgaatgagagttatctggtcatcaatatgtaagttMNNaaaacctggcaatgatttagaccacttgactactgaaagaag	-2.6

Gene	Target	ssODN	Sequence	ΔG
PGR	W755	oST_ZPM_W211X-T10250N_G10251N_G10252K	ccaaacaccattaagctcatMNNagaatactgaatgagagttatctggtcatcaatatgtaagtttcgaaaacctggcaatgatttagac	-4.4
PGR	M756	oST_ZPM_M212X-A10253N_T10254N_G10255K	agaccaaacaccattaagctMNNccaagaatactgaatgagagttatctggtcatcaatatgtaagtttcgaaaacctggcaatgattta	-5.7
PGR	M759	oST_ZPM_M215X-A10262N_T10263N_G10264K	ctccatcctagaccaaacacMNNtaagctcatccaagaatactgaatgagagttatctggtcatcaatatgtaagtttcgaaaacctggc	-3.7
PGR	V760	oST_ZPM_V216X-G10265N_T10266N_G10267K	gatctccatcctagaccaaaMNNcattaagctcatccaagaatactgaatgagagttatctggtcatcaatatgtaagtttcgaaaacct	-3.7
PGR	L763	oST_ZPM_L219X-C10274N_T10275N_A10276K	gtgcaaaatacagcatctgcccactgacatgtttgtaggatctccatccMNNaccaaacaccattaagctcatccaagaatactgaatga	-4.5
PGR	R766	oST_ZPM_R222X-A10283N_G10284N_A10285K	gtgcaaaatacagcatctgcccactgacatgtttgtaggaMNNccatcctagaccaaacaccattaagctcatccaagaatactgaatga	-6.7
PGR	R766	oST_ZPM_R222X-A10283N_G10284N_A10285K	gtgcaaaatacagcatctgcccactgacatgtttgtaggaMNNccatcctagaccaaacaccattaagctcatccaagaatactgaatga	-6.7
PGR	F778	oST_ZPM_F234X-T10319N_T10320N_T10321K	atgatgattctttcatccgctgttcatttagtattagatcaggtgcMNNatacagcatctgcccactgacatgtttgtaggatctccatc	-5.8
PGR	F794	oST_ZPM_F250X-T10367N_T10368N_C10369K	tggctaacttgaagcttgacaaactcctgtgggatctgccacatggtaaggcataatgaataMNNtgatgattctttcatccgctgttca	-7.5
PGR	L797	oST_ZPM_L253X-T10376N_T10377N_A10378K	tgggatctgccacatggtaaggcaMNNtgaatagaatgatgattctttcatccgctgttcatttagtattagatcaggtgcaaaatacag	-7.1
PGR	M801	oST_ZPM_M257X-A10388N_T10389N_G10390K	tacagaggaactcttcttggctaacttgaagcttgacaaactcctgtgggatctgccaMNNggtaaggcataatgaatagaatgatgatt	-5
PGR	R836	oST_ZPM_R292X-C10493N_G10494N_A10495K	taatgtagcttgacctcatctcctcaaactgggtttgactMNNtagcccttccaaaggaattgtattaagaagtaacaatactttcatac	-6.6
PGR	R869	oST_ZPM_R325X-C10592N_G10593N_T10594K	aagctgtttgacaagatcatgcaagttatcaagaagttttgtaagttgatagaaMNNctgtgagctcgacacaactccttttgcctcaa	-4.5
PGR	L887	oST_ZPM_L343X-C10646N_T10647N_T10648K	atttctggaaattcaacactcagtgcccgggactggataaatgtattcaagcagtacagatgMNNctgtttgacaagatcatgcaagtta	-4.6
PGR	Y890	oST_ZPM_Y346X-T10655N_A10656N_C10657K	gacatcatttctggaaattcaacactcagtgcccgggactggataaatgtattcaagcaMNNcagatgaagctgtttgacaagatcatgc	-3.2
PGR	Y890	oST_ZPM_Y346X-T10655N_A10656N_C10657K	gacatcatttctggaaattcaacactcagtgcccgggactggataaatgtattcaagcaMNNcagatgaagctgtttgacaagatcatgc	-3.2
PGR	Y890	oST_ZPM_Y346C-A10656G	atttctggaaattcaacactcagtgcccgggactggataaatgtattcaagcagCacagatgaagctgtttgacaagatcatgcaagtta	-8
PGR	C891	oST_ZPM_C347X-T10658N_G10659N_C10660K	atttctggaaattcaacactcagtgcccgggactggataaatgtattcaaMNNgtacagatgaagctgtttgacaagatcatgcaagtta	-4.2
PGR	T894	oST_ZPM_T350X-A10667N_C10668N_A10669K	agcaataacttcagacatcatttctggaaattcaacactcagtgcccgggactggataaaMNNattcaagcagtacagatgaagctgttt	-4.4
PGR	R899	oST_ZPM_R355X-C10682N_G10683N_G10684K	tgcagcaataacttcagacatcatttctggaaattcaacactcagtgcMNNggactggataaatgtattcaagcagtacagatgaagctg	-3.9
PGR	F905	oST_ZPM_F361X-T10700N_T10701N_T10702K	tgcagcaataacttcagacatcatttctggMNNttcaacactcagtgcccgggactggataaatgtattcaagcagtacagatgaagctg	-3.9
PGR	M909	oST_ZPM_M365X-A10712N_T10713N_G10714K	tgtgcagcaataacttcagaMNNcatttctggaaattcaacactcagtgcccgggactggataaatgtattcaagcagtacagatgaagc	-3.6

46

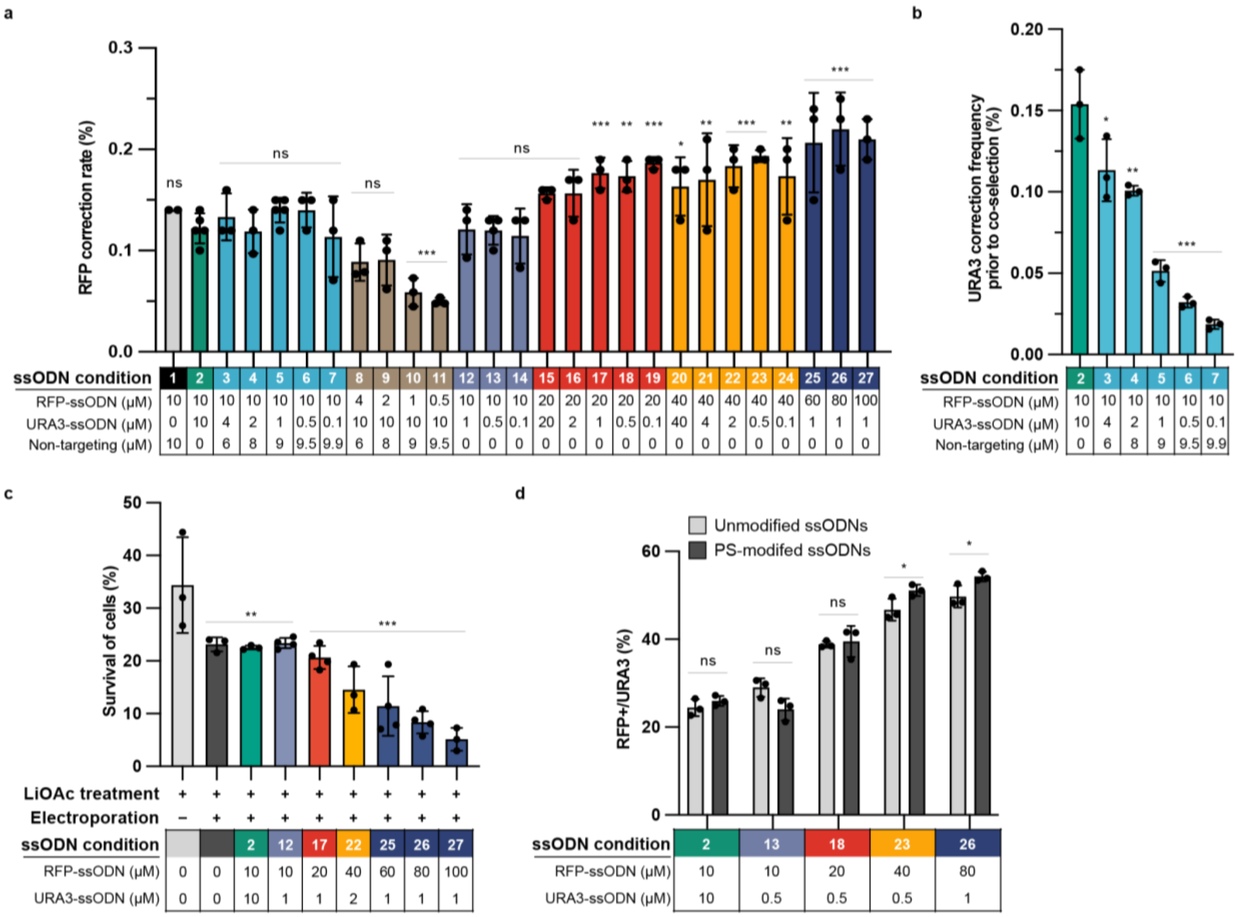

Supplementary Figure 1: Effect of ssODN concentration and modification on frequency of edited cells and cell viability. a, Frequency of RFP positive cells in ssODN-transformed population prior to -Ura selection under different ssODN concentrations and ratios used in **Fig. 1e**. It positively correlates with the concentration of RFP-ssODN. **b**, Frequency of URA3 cells decreases with URA3-ssODN concentration. **c**, Cell survival of selective conditions used in **Fig. 1e**, showing decreased viability after electroporation with increased ssODN concentration. Cell viability data were normalized to untreated cells (100 % viable). **d**, Protecting ssODN from exonuclease degradation by modifying the last 4 nucleotides of its 5' and 3' ends with phosphorothioate bonds does not result in major enhancement of eMAGE ARF as compared to unprotected ssODNs. All values represent mean ± SD for at least three replicates. p values of multiple-group comparisons from ordinary one-way ANOVA Dunnett's test and p values of two-group comparisons from unpaired t-test. ns, not significant, *$p < 0.05$, **$p < 0.01$, ***$p < 0.001$.

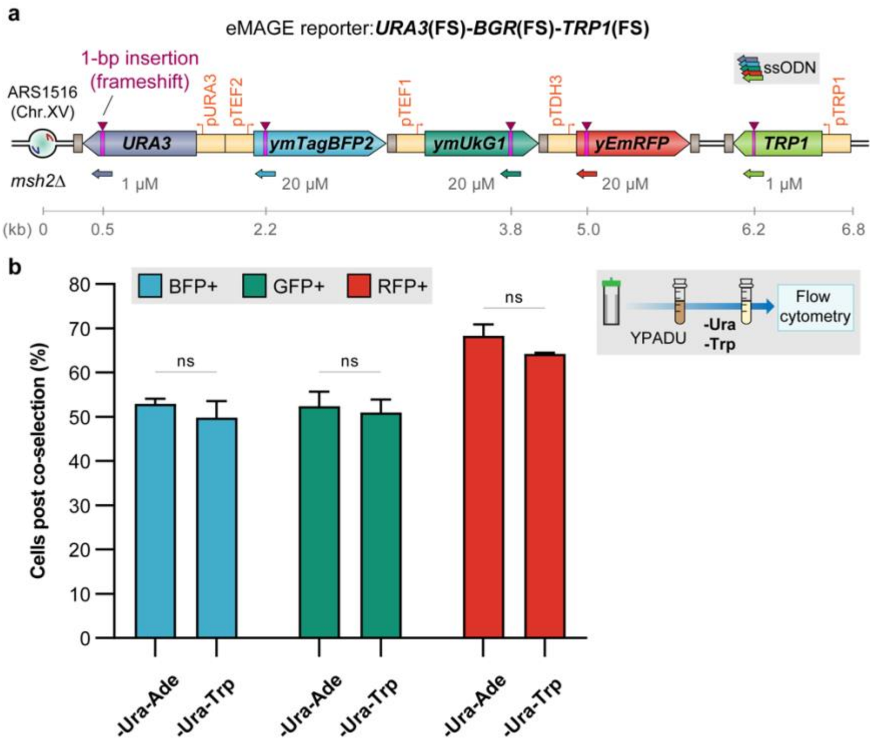

Supplementary Figure 2: Comparison of dual marker co-selection using ADE2 vs TRP1 as a second marker. a, Genetic architecture of an eMAGE reporter with an alternative set of co-selection markers. b, Genome editing efficiency post co-selection, selecting with either -Ura, -Ade media or -Ura, -Trp media. All values represent mean ± SD for at least three replicates. Statistical significance analysis of the means of each of the editing categories was performed. p values from ordinary one-way ANOVA Dunnett's test. ns, not significant.

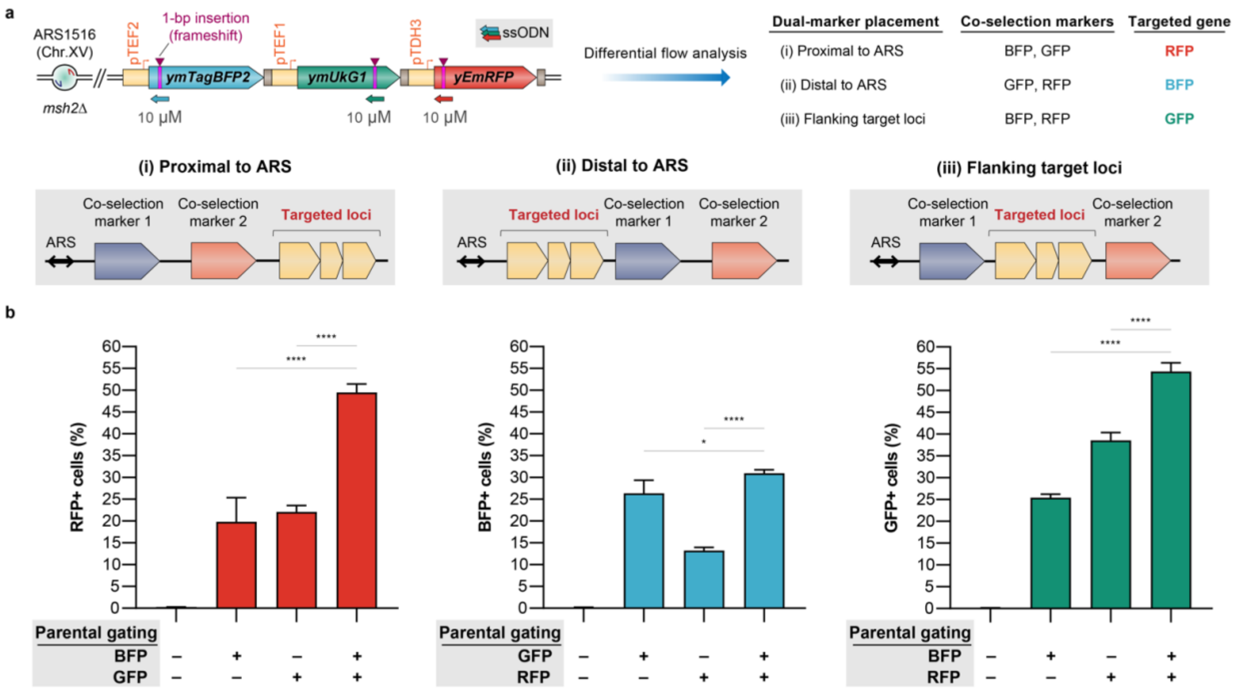

Supplementary Figure 3: Comparison of three dual-marker placement strategies. a, eMAGE reporter used in this analysis containing the same frameshifted fluorescent reporter genes as the one in **Fig. 2a**. To simulate the three possible architectures for co-selection marker placement: (i) proximal to ARS, (ii) distal to ARS, and (iii) flanking targeted loci, the flow cytometry data collected from the same experiments were analyzed as follows: two of the three fluorescent markers were used as parental gates to select the cells with either single or double positive fluorescent phenotypes. The percentage of these gated cells that are positive for the remaining (third) fluorescent gene represents the eMAGE co-selection ARF. **b**, Comparison of eMAGE ARF using co-selection with either a single or dual fluorescent marker in the above three placement configurations. In all scenarios, co-selection with two markers (last bar) yields higher eMAGE ARF compared to co-selection with only a single marker. Architecture (iii), in which the co-selection markers flank the targeted loci, resulted in the highest ARF gain among the tested dual-marker placement strategies. All values represent mean ± SD for at least three replicates. p values from ordinary one-way ANOVA Dunnett's test. *p < 0.05, **p < 0.01, ***p < 0.001, ****p < 0.0001.

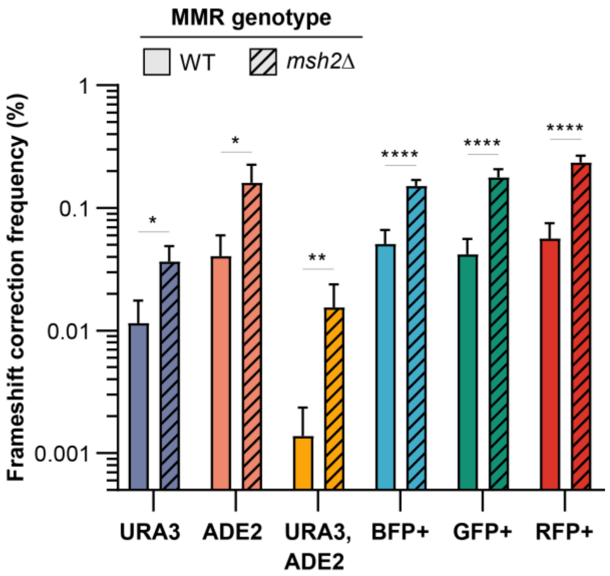

Supplementary Figure 4: MMR deficiency promotes eMAGE editing and benefits implementation of dual-marker co-selection. a, Comparison of MMR wild-type (WT) and *MSH2* knockout (*msh2*Δ) strains for their frameshift correction frequency of each marker in the eMAGE reporter shown in **Fig. 2a**. Deletion of *MSH2* confers a three to five-fold increase in frameshift correction frequency and a 10-fold increase of double-edited URA3, ADE2 cells. All values represent mean ± SD for at least three replicates. p values from unpaired t-test. $^{*}p < 0.05$, $^{**}p < 0.01$, $^{***}p < 0.001$, $^{****}p < 0.0001$.

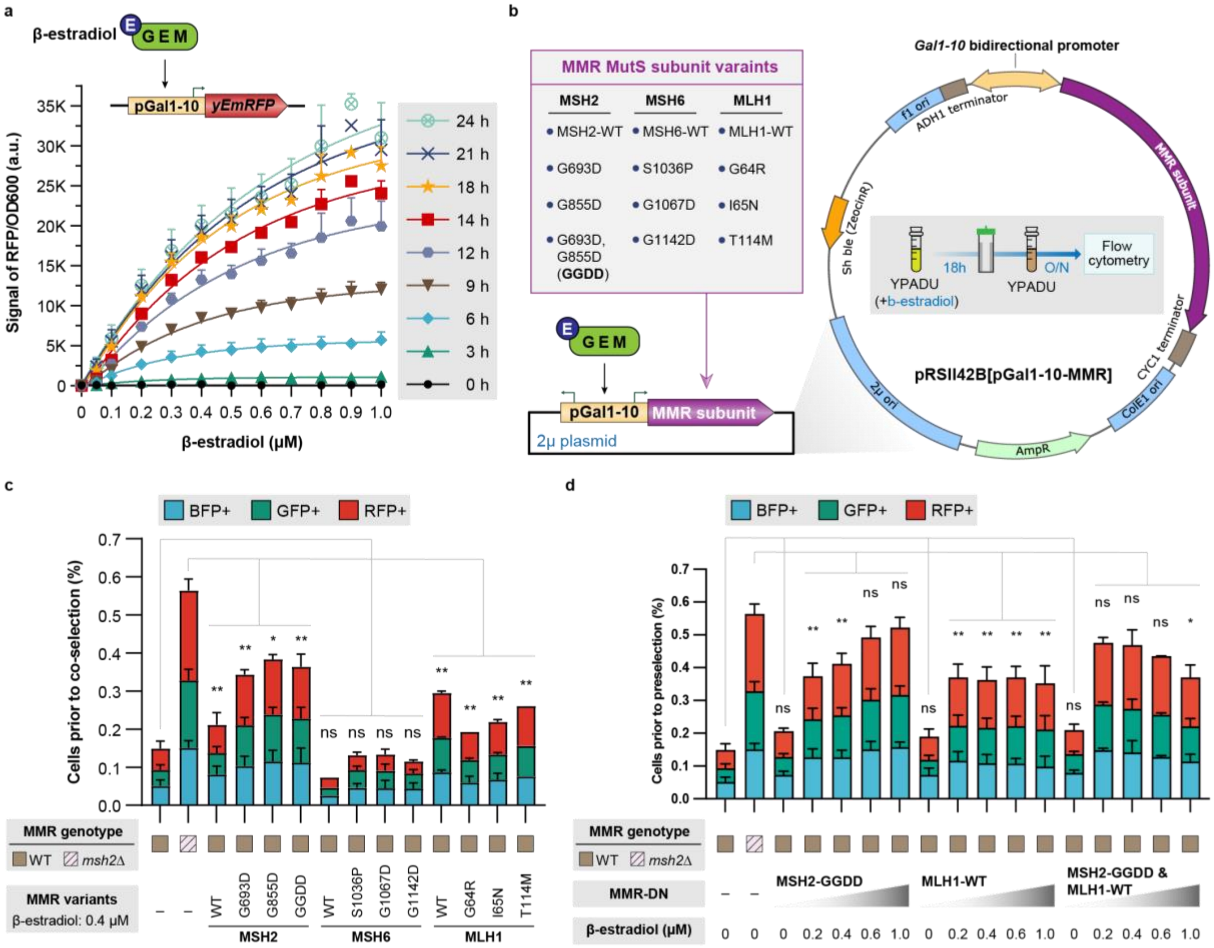

Supplementary Figure 5: Engineering tunable expression of dominant negative mutants to modulate MMR fidelity during eMAGE editing. a, RFP induction over 24 hours with titration of β-estradiol concentration reveals a robust dose-response and time-dependent expression curve. **b**, Selected MMR subunit variants of three wild-type proteins and nine dominant negative mutants overexpressed using our improved β-estradiol inducible system from high-copy 2μ plasmids (see plasmids used in this study in **Supplementary Table 4**). This vector allows inducible expression of up to two MMR subunits simultaneously using the bidirectional *Gal1-10* promoter upon supplying β-estradiol in growth media of strains with GEM expression. Expression of subunit variants from episomal vector was induced with β-estradiol for 18 hours prior to ssODN electroporation. After electroporation cells were recovered overnight in medium without β-estradiol and analyzed by flow cytometry without co-selection**.** Notably**,** this vector is also compatible with conventional galactose induction providing flexibility for yeast strains without a pre-integrated GEM cassette. However, galactose induction is not preferable due to the

expression level of MMR-DN is more difficult to be controlled and it requires carbon source changes that could perturb cell growth and increase protocol time. **c**, Influence of 12 MMR subunit variants on ssODN-mediated frameshift correction frequency in three fluorescent reporter genes shown in **Fig. 2a,** as compared to MMR-proficient (WT) and MMR-deficient (*msh2*Δ) cells. Expression of subunit variants from episomal vector was induced with 0.4 μM β-estradiol for 18 hours (~50% of maximum overexpression) prior to ssODN electroporation. **d**, Modulation of MMR fidelity by titrating MSH2-GGDD and MLH1-WT expression with different β-estradiol concentrations. p values of multiple-group comparisons from ordinary one-way ANOVA Dunnett's test and p values of two-group comparisons from unpaired t-test. ns, not significant, $^{*}p < 0.05$, $^{**}p < 0.01$, $^{***}p < 0.001$.
